# Computational Structural Comparison of Toxoplasma gondii CDPK1 and Human BUB1 kinases: Implications for Selective Inhibitor Design

**DOI:** 10.1021/acsomega.5c00640

**Published:** 2025-06-11

**Authors:** João Pedro Bezerra Carvalho, Deborah Antunes, Daniel Adesse, Ana Carolina Guimarães

**Affiliations:** † Laboratory for Applied Genomics and Bioinnovations, Oswaldo Cruz Institute (IOC - FIOCRUZ), Rio de Janeiro 21040-900, Brazil; ‡ Structural Biology laboratory, Oswaldo Cruz Institute (IOC - FIOCRUZ), Rio de Janeiro 21040-900, Brazil; § Laboratory of Ocular Immunology and Transplantation, Bascom Palmer Eye Institute, Miller School of Medicine, University of Miami, Miami Florida 33136, United States

## Abstract

Toxoplasmosis, affecting one-third of the global human population, urgently requires new therapeutic strategies due to current treatment limitations and drug resistance. Toxoplasma gondii calcium-dependent protein kinase 1 (TgCDPK1) is a promising drug target due to its essential role in parasite survival and its presumed unique glycine gatekeeper residue. Aiming at identifying new and selective inhibitors of TgCDPK1, we performed computational structural analyses and unexpectedly found that a human kinase (BUB1), despite having only 14% sequence identity, shares this glycine gatekeeper with TgCDPK1, which initially raised concerns for selective inhibitor development. Subsequent analyses revealed distinct electrostatic properties and binding site architectures between these kinases. Molecular dynamics simulations demonstrate differential binding pocket dynamics, with TgCDPK1 showing more focused interaction networks compared to BUB1’s dispersed patterns. Virtual screening of apicomplexan kinase inhibitors confirms stronger binding affinity for TgCDPK1 over BUB1, supporting the continued development of selective therapeutics against toxoplasmosis.

## Graphical abstract

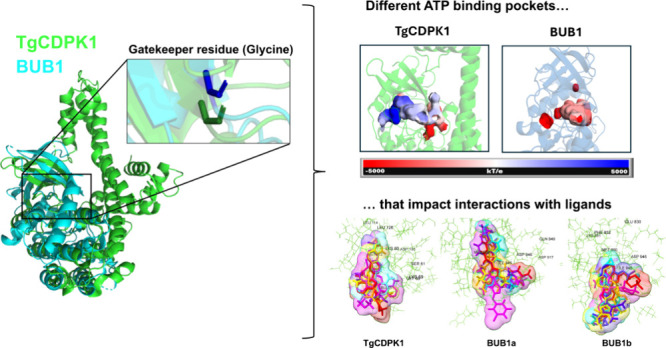

## Introduction

Toxoplasmosis, caused by the obligate intracellular protozoan parasite Toxoplasma gondii, is a globally prevalent infection affecting approximately one-third of the world’s population.^1^ While often asymptomatic in immunocompetent individuals, it can lead to severe complications in immunocompromised patients and congenitally infected newborns.^2,3^ Current therapeutic options for toxoplasmosis, primarily based on pyrimethamine and sulfadiazine combinations, are limited by significant side effects, poor tolerability, and the potential for drug resistance.^4,5^ These limitations underscore the urgent need for novel, more effective, and better-tolerated treatments.

In recent years, the calcium-dependent protein kinase 1 of T. gondii (TgCDPK1) has emerged as a promising drug target.^6,7^ TgCDPK1 plays a crucial role in the parasite’s life cycle, regulating processes such as host cell invasion, egress, and motility.^8,9^ A unique structural feature of TgCDPK1 is the presence of a glycine residue (Gly128) at the gatekeeper position within its ATP-binding pocket.^10^ This small amino acid creates an enlarged hydrophobic pocket not typically found in human kinases,^11,12^ in which the gatekeeper position is typically occupied by larger amino acids such as phenylalanine, threonine, or methionine.^13,14^ This distinctive structural feature has enabled the development of bumped kinase inhibitors (BKIs), which have shown promising antiparasitic activity and selectivity.^15,16^

Several studies have demonstrated the potential of targeting TgCDPK1 for therapeutic intervention. Ojo et al. (2010) reported the first series of selective TgCDPK1 inhibitors, demonstrating their efficacy in blocking T. gondii growth in vitro using human fibroblast cultures.^10^ Subsequently, Vidadala et al. (2016) developed orally available TgCDPK1 inhibitors with improved pharmacokinetic properties and demonstrated their ability to reduce brain cyst burden in a mouse model of chronic toxoplasmosis.^17^ These studies highlight the promise of TgCDPK1 as a drug target but also highlight the need for a deeper understanding of its structural uniqueness compared to human kinases.

Despite previous assumptions, the presence of a glycine gatekeeper in parasite kinases and its potential occurrence in human kinases requires systematic investigation. A comprehensive structural analysis of human kinases, particularly focusing on the gatekeeper residue and ATP-binding pocket, is essential for the rational design of truly selective inhibitors. Such an analysis is crucial to avoid potential off-target effects and to ensure the development of safe and effective therapeutics.^18^

This study combines sequence- and structure-based approaches to screen the human kinome for potential TgCDPK1 structural homologues. Our analysis focuses on comparing the ATP-binding pocket architecture and gatekeeper region between TgCDPK1 and human kinases, including their interaction patterns with the known inhibitor UW2 ^17^

This structural comparison between TgCDPK1 and human kinases provides essential insights into designing selective inhibitors against toxoplasmosis. Our findings establish a framework for structure-guided drug discovery that optimizes both efficacy and safety in antiparasitic drug development.

## Results

### Identification of BUB1 as a Human Kinase with a Glycine Gatekeeper

To evaluate the feasibility of developing selective inhibitors targeting TgCDPK1, a comprehensive analysis of the human kinome revealed ten protein kinases sharing significant sequence similarity with TgCDPK1’s kinase domain (residues 51–308). These kinases included Checkpoint Kinase 2 (CHK2), serine/threonine-protein kinase H2 (KPSH2), calcium/calmodulin-dependent protein kinase type 1A (KCC1A), serine/threonine-protein kinase H1 (KPSH1), calcium/calmodulin-dependent protein kinase type 1D (KCC1D), salt-inducible kinase 2 (SIK2), myosin light chain kinase family member 4 (MYLK4), Ribosomal protein S6 kinase alpha-5 (KS6A5), calcium/calmodulin-dependent protein kinase type 1G (KCC1G), and MAP/microtubule affinity-regulating kinase 1 (MARK1). These kinases exhibited sequence identity between 40.4 and 45.2% with coverage ranging from 42.8 to 51.5% (Table 1 and Figure S1). Despite their sequence similarity to that of TgCDPK1, none of these kinases possessed the characteristic glycine gatekeeper residue. However, considering that proteins with low sequence identity may still share significant structural similarities,^19^ we hypothesized that a structure-based approach might reveal human kinases with this key feature that were missed by sequence-based methods.

**Table 1:** Sequence Analysis of Human Protein Kinases Identified through the BLASTp Search against TgCDPK1^a^

**sequence**	**identity (%)**	**coverage (%)**	**length (residues)**	***qstart***	***qend***	***sstart***	***ssend***	***E-value***	***score***
CHK2	41.328	51,48	271	49	309	218	487	5.25 × 10^–59^	2713
KPSH2	40.530	51,48	264	50	310	62	322	9.64 × 10^–58^	2638
KCC1A	42.912	51,28	261	49	308	18	276	3.21 × 10^–66^	2627
KPSH1	41.509	51,08	265	50	308	97	355	1.18 × 10^–60^	2535
KCC1D	41.699	50,89	259	51	308	23	279	1.80 × 10^–^^64^	2516
SIK2	40.385	50,89	260	51	308	20	271	3.27 × 10^–^^53^	2397
MYLK4	40.541	50,49	259	56	311	111	364	4.37 × 10^–56^	2202
KS6A5	42.264	50,30	265	55	309	430	688	3.80 × 10^–56^	2187
KCC1G	44.882	49,90	254	56	308	28	277	9.36 × 10^–65^	2185
MARK1	45.205	42,80	219	51	267	60	274	1.21 × 10^–52^	2152

a Parameters: qstart and qend indicate the start and end positions of the aligned region in the query sequence (TgCDPK1); sstart and ssend represent the start and end positions in the subject sequences (human kinases); *E*-value indicates the statistical significance of the alignment, with lower values suggesting higher significance; score represents the quality of the alignment, with higher values indicating better matches. Sequence identity and coverage percentages demonstrate the degree of similarity and extent of alignment between TgCDPK1 and human kinases, respectively.

This hypothesis proved correct when structure-based screening using the DALI server^20^ identified human BUB1 (Mitotic checkpoint serine/threonine-protein kinase BUB1) as possessing a glycine residue at the gatekeeper position. This finding was supported by both crystallographic (6F7B) and predicted (AlphaFold2:^21^ AF-O43683) structures, which showed RMSD values of 4,8 and 3,6 Å, respectively, when aligned with TgCDPK1’s kinase domain, despite sharing only 14% sequence identity. Among 2010 structures analyzed (1350 from the Protein Data Bank^22^ and 660 from the AlphaFold database), BUB1 was the only human kinase identified with this critical feature.

Structural searches were also performed using the FoldSeek platform,^23^ examining multiple databases: PDB100, AF-DB proteome, AF-DB50, and AF-DB Swissprot. Among 793 human proteins identified in the PDB100 database, only one structure contained a glycine gatekeeper residue, a DAPK1 protein with an experimental L to G mutation, rather than a naturally occurring feature. Analysis of the AF-DB Swissprot database identified 99 human proteins, the AF-DB proteome database yielded 77 human proteins, and no human proteins were found in the AF-DB50 database. Notably, none of these proteins naturally possessed a glycine gatekeeper residue, further confirming BUB1’s uniqueness among human kinases.

BUB1, which regulates mitotic checkpoint signaling,^24^ is expressed across various human tissues, including the thymus, colon, spleen, lung, and small intestine.^25^ The identification of this widely expressed human kinase sharing TgCDPK1’s glycine gatekeeper has significant implications for inhibitor design strategies, as most TgCDPK1 inhibitors exploit this key structural feature. To understand the potential impact of this discovery on drug development efforts, we performed detailed sequence and structural comparisons between these two kinases.

### Comparative Analysis of TgCDPK1 and BUB1 Binding Site Architecture

Following the identification of BUB1 as a human kinase containing a glycine gatekeeper, a detailed comparative analysis between TgCDPK1 and BUB1 revealed both conserved architectural features and distinctive characteristics that could impact selective inhibitor design (Figure 1B,C). Initial examination through global alignment revealed high conservation of ATP-binding pocket residues (Figure 1A–E) with both kinases sharing identical glycine positions (Gly128 in TgCDPK1 and Gly937 in BUB1) and highly preserved catalytic sites. The hinge region demonstrated remarkable structural similarity, with only a subtle variation - a valine residue in TgCDPK1 and a leucine in BUB1.

**Figure 1:**
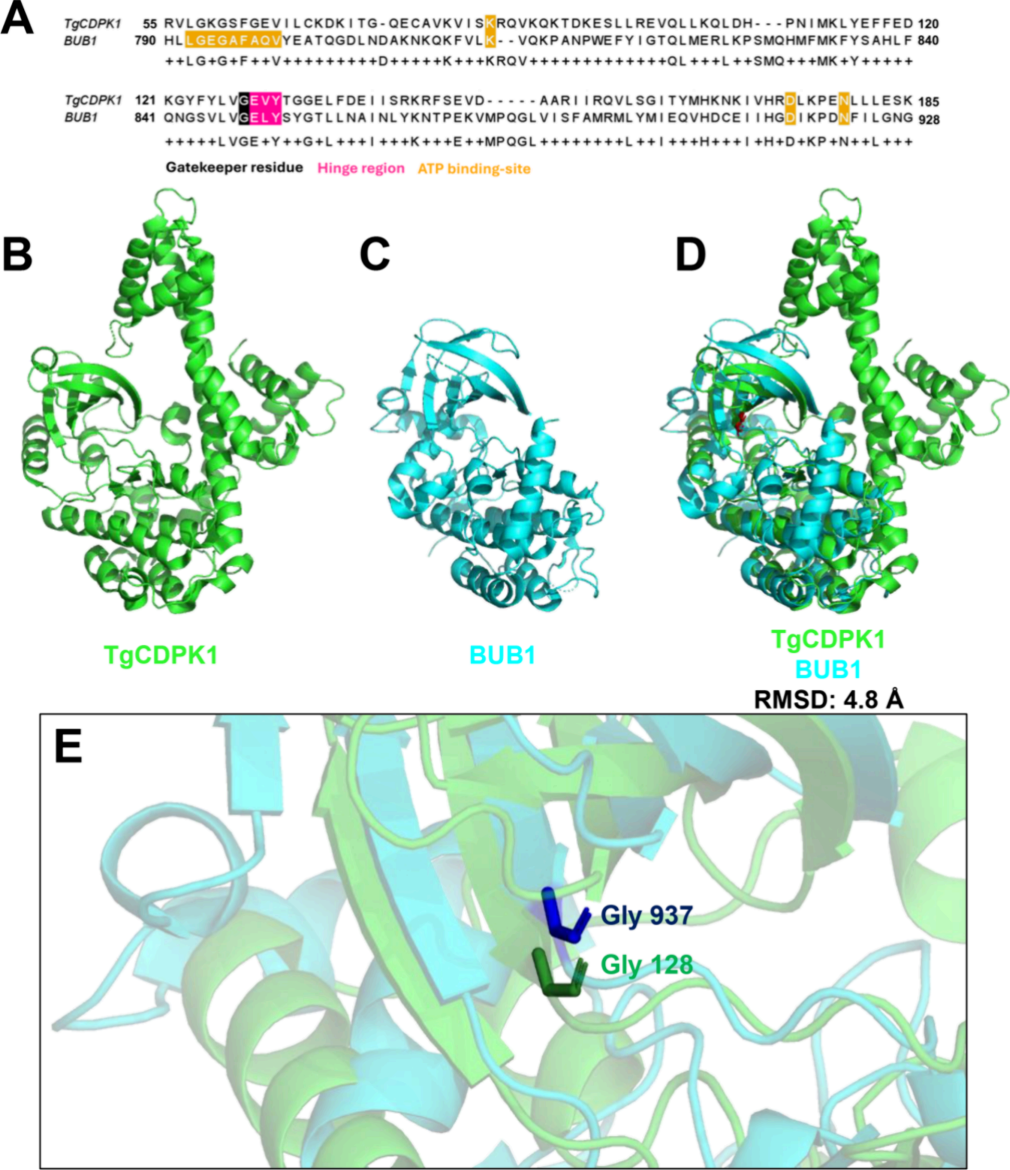
Comparison of TgCDPK1 (3I7B) and BUB1 (6F7B) kinases. (A) Sequence alignment highlighting the ATP binding site (orange), glycine gatekeeper residue (black), and hinge region (pink). (B) TgCDPK1 structure. (C) BUB1 structure. (D) Structural alignment (RMSD: 4.8 Å) showing the bilobal kinase fold organization (gatekeeper residues in red). (E) Zoom of the ATP binding-pocket with Gly128 (TgCDPK1) highlighted in green and Gly937­(BUB1) highlighted in blue.

To better understand the three-dimensional organization of these kinases, the structural alignment of their kinase domains showed an RMSD of 4.8 Å (Figure 1D). Both kinases exhibit the characteristic bilobal kinase fold, comprising a smaller N-terminal lobe dominated by β-sheets and a larger C-terminal lobe rich in α-helices. While this overall architecture is typical of protein kinases,^26^ detailed examination of their binding pockets revealed notable distinctions.

A thorough analysis of the ATP-binding pocket reveals both conserved elements and critical structural differences between TgCDPK1 and BUB1. Both kinases share key residues typical for nucleotide binding, including a lysine (Lys80 in TgCDPK1; Lys821 in BUB1) that coordinates with ATP’s phosphate groups, residues forming the glycine-rich loop, and a glutamate in the hinge region (Glu129 in TgCDPK1; Glu867 in BUB1).^25^ However, significant differences exist in their structure and catalytic mechanisms. BUB1 exhibits a distinctive metal coordination network involving a magnesium ion that interacts with Asp946,^30,31^ which is absent in the TgCDPK1 structure. Additionally, BUB1 contains Ser969 in its P + 1 loop, which undergoes regulatory phosphorylation critical for its activation.^27^ A major structural distinction is that TgCDPK1 possesses a calcium-dependent regulatory domain with EF-hand motifs (residues 316–507) that is completely absent in BUB1.^10,11^ This domain is responsible for calcium-mediated activation of TgCDPK1, representing a fundamentally different regulatory mechanism compared to BUB1’s phosphorylation-dependent activation.^12^ These structural and mechanistic distinctions highlight the fundamental differences between these kinases, despite their shared glycine gatekeeper feature.

Close examination of the ATP-binding pockets revealed striking similarities in the gatekeeper region (Figure 2A,B). As expected from sequence analysis, the presence of glycine at this position (Gly128 in TgCDPK1 and Gly937 in BUB1) creates an enlarged hydrophobic pocket adjacent to the ATP-binding site in both kinases (red circles in Figure 2A,B). This feature is particularly significant, as it markedly contrasts with other human kinases identified, exemplified by CHK2, where bulkier gatekeeper residues restrict access to this region (Figure 2C).

**Figure 2:**
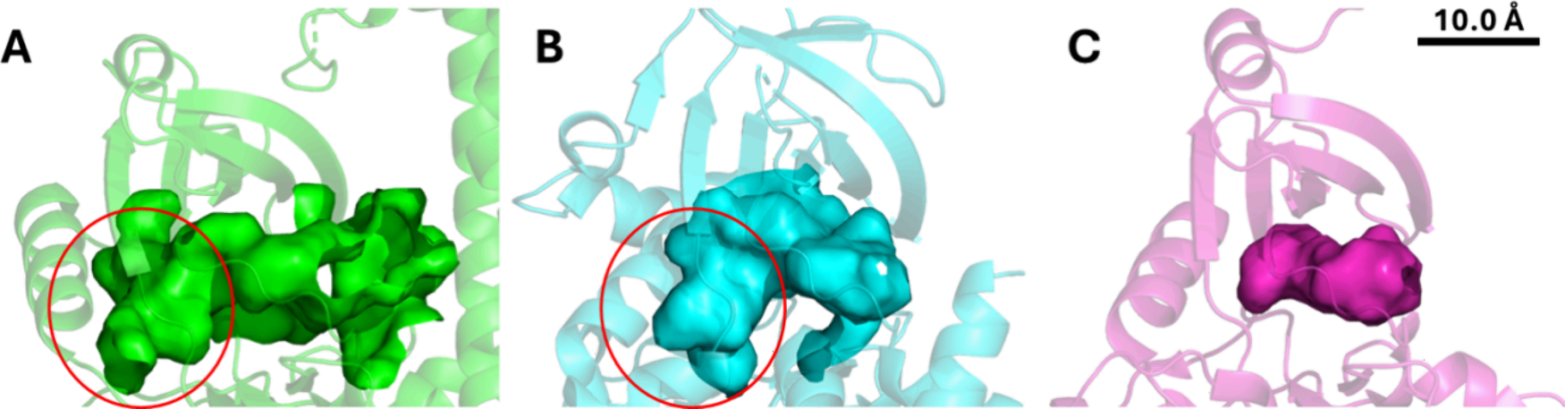
ATP-binding pockets of three kinases. (A) TgCDPK1 with the enlarged hydrophobic subpocket (red circle) created by Gly128. (B) BUB1 showing a similar extra subpocket due to Gly937. (C) CHK2 lacking the additional subpocket due to its bulkier gatekeeper residue.

Quantitative analysis of binding site topology using DogSite3 ^27–29^ demonstrated comparable pocket architectures between TgCDPK1 and BUB1, with volumes of 512.51 and 566.03 Å^3^, respectively (Table 2). These volumes substantially exceed those of other analyzed human kinases, which range from 74.75 to 335.87 Å^3^. Similarly, both kinases exhibit deeper binding pockets (TgCDPK1:18.71 Å; BUB1:18.12 Å) compared to other human kinases (range: 7.55–13.83 Å).

**Table 2:** Volumetric and Depth Analyses of ATP-Binding Cavities from TgCDPK1 and Human Kinases^a^

**protein**	**volume (Å** ^**3**^ **)**	**depth (Å)**
TgCDPK1	512.51	18.71
BUB1	566.03	18.12
CHK2	335.87	13.62
KCC1A	74.75	8.50
KCC1D	93.18	7.55
KCC1G	155.65	9.93
KPSH1	205.82	10.31
KPSH2	194.05	11.23
KS6A5 C-terminal	196.61	12.65
KS6A5 N-terminal	187.90	11.57
MARK1	147.97	9.26
MYLK4	272.38	13.83
SIK2	238.08	14.13

a Parameters: Volume (Å^3^) represents the calculated spatial capacity of the ATP-binding cavity as determined by DogSite3 analysis; depth (Å) indicates the maximum distance from the cavity entrance to its deepest point.

Despite these shared characteristics, structural alignment identified 11 residues in the TgCDPK1 ATP-binding pocket without direct spatial correspondence in BUB1: M165, D174, L198, A204, S205, K209, D210, K211, I212, T214, and I218 (Figure 3). Furthermore, electrostatic surface analysis revealed distinct charge distributions within the binding pockets. While TgCDPK1 displays a mixed electrostatic character with both positive and negative regions, BUB1 exhibits predominantly negative potential (Figures 3C and S1). These differences in residue composition and electrostatic properties suggest potential avenues for achieving selective inhibition despite the shared glycine gatekeeper feature.

**Figure 3:**
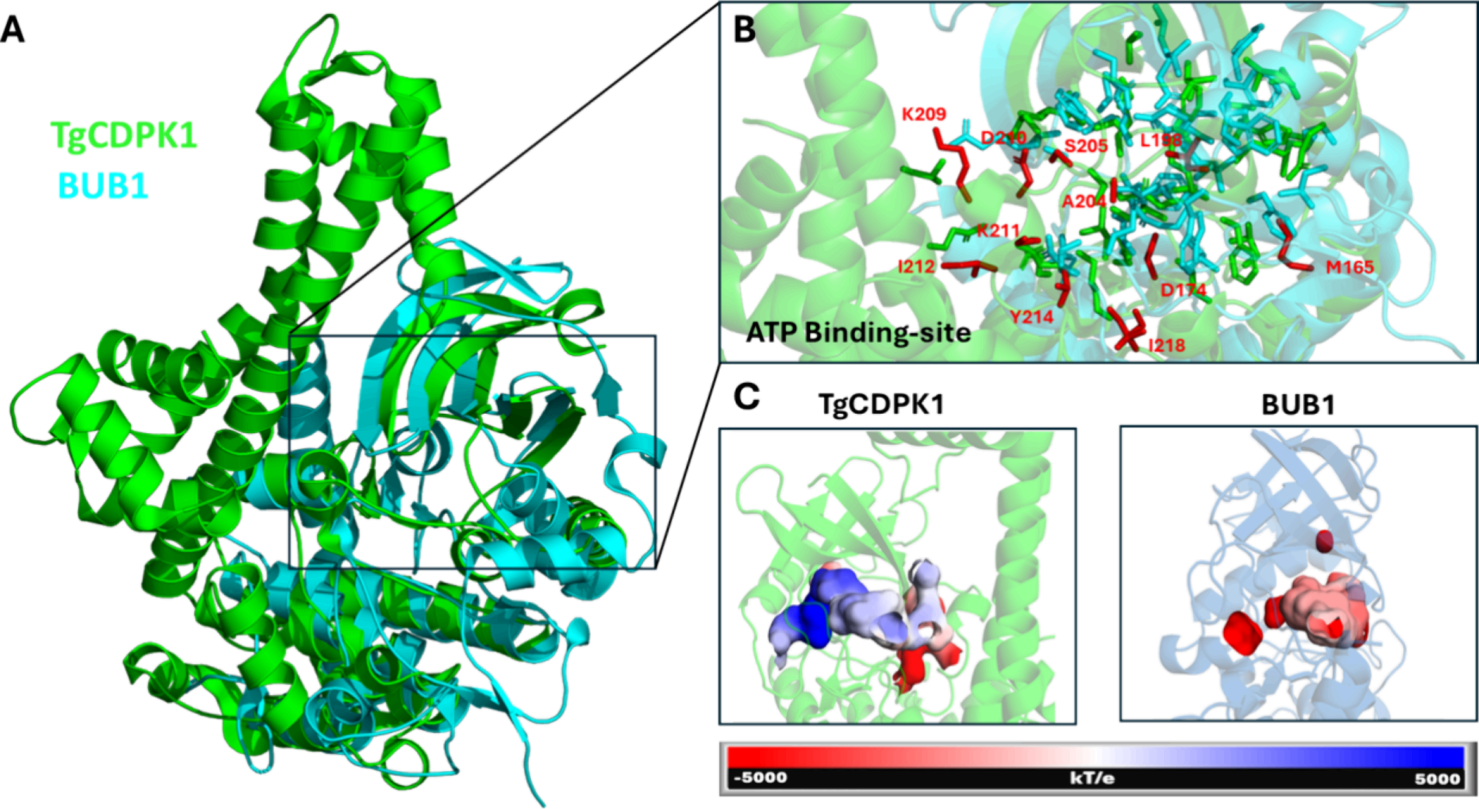
ATP-binding site comparison between TgCDPK1 and BUB1. (A) Structural alignment with TgCDPK1 (green) and BUB1 (cyan). (B) Close-up view highlighting colocalized residues (green/cyan) and TgCDPK1 residues without corresponding positions in BUB1 (red). (C) Electrostatic potential maps showing TgCDPK1’s mixed character (left) versus BUB1’s predominantly negative potential (right). Scale: −5000 kT/e (red) to +5000 kT/e (blue).

### Dynamics of TgCDPK1 and BUB1 Binding Sites Revealed by Molecular Simulations

Building upon our structural analyses and to understand the dynamic behavior of these kinases, we sought to investigate the dynamic behavior of both kinases in complex with biologically relevant ligands. For TgCDPK1, we selected the complex with UW2, a well-characterized selective inhibitor that exploits the glycine gatekeeper feature.^17^ For BUB1, we examined two distinct scenarios: its interaction with ADP (the product of ATP hydrolysis) with Ser969 in its phosphorylated state, and its complex with BAY 1816032 (CWQ, a selective inhibitor of BUB1)^30^ where Ser969 remains unphosphorylated. This selection strategy allowed us to compare both the natural catalytic behavior in a phosphorylation-dependent state and the inhibitor-bound configuration of these kinases.^31^ We performed 2 replicates of extensive molecular dynamics simulations of these complexes over 1 μs trajectories.

All simulated complexes demonstrated stable trajectories over the 1 μs time scale, with distinct stability profiles between TgCDPK1 and BUB1 systems (Figure S2). The TgCDPK1 complexed with UW2 exhibited moderate heavy atom flexibility (RMSD = 3.27 ± 0.46 Å), with the UW2 inhibitor maintaining a relatively consistent binding pose (RMSD = 2.49 ± 0.71 Å) despite showing the highest ligand fluctuations among all complexes. The BUB1 complexed with CWQ displayed the most stable configuration, with both protein (RMSD = 2.68 ± 0.3 Å) and ligand (RMSD = 1.02 ± 0.21 Å) maintaining remarkably low deviations throughout the simulation. The BUB1 complexed with ADP showed intermediate stability, with protein backbone fluctuations (RMSD = 3.25 ± 0.32 Å) comparable to those of TgCDPK1 complexed with UW2, but more constrained ligand mobility (RMSD = 2.12 ± 0.41 Å).

Notably, all systems reached equilibrium within the first 500 ns, after which they maintained stable conformational ensembles. The higher ligand RMSD observed in TgCDPK1 complexed with UW2 suggests greater conformational sampling of the binding pocket, which could be relevant for understanding inhibitor selectivity. In contrast, the exceptional stability of CWQ in the BUB1 binding pocket indicates a highly optimized fit for this selective inhibitor.

To gain deeper insights into local protein dynamics, RMSF data analysis reveals distinctive patterns of structural flexibility in kinase proteins. In TgCDPK1 (Figure 4A), the highest fluctuations were observed in the N-terminal loop region (residues 40–50), with RMSF values exceeding 6.0 Å, indicating substantial structural flexibility. Additional peaks in flexibility were noted at residues 320–330, 370–380, 420–430, and 495–500, corresponding to surface-exposed loops and terminal regions. In contrast, the central domain of TgCDPK1, particularly residues 100–300, exhibited low RMSF values (<2.0 Å), suggesting a stable core. Notably, this region encompasses the ATP-binding pocket, indicating that ligand binding contributes to local structural rigidity.

**Figure 4:**
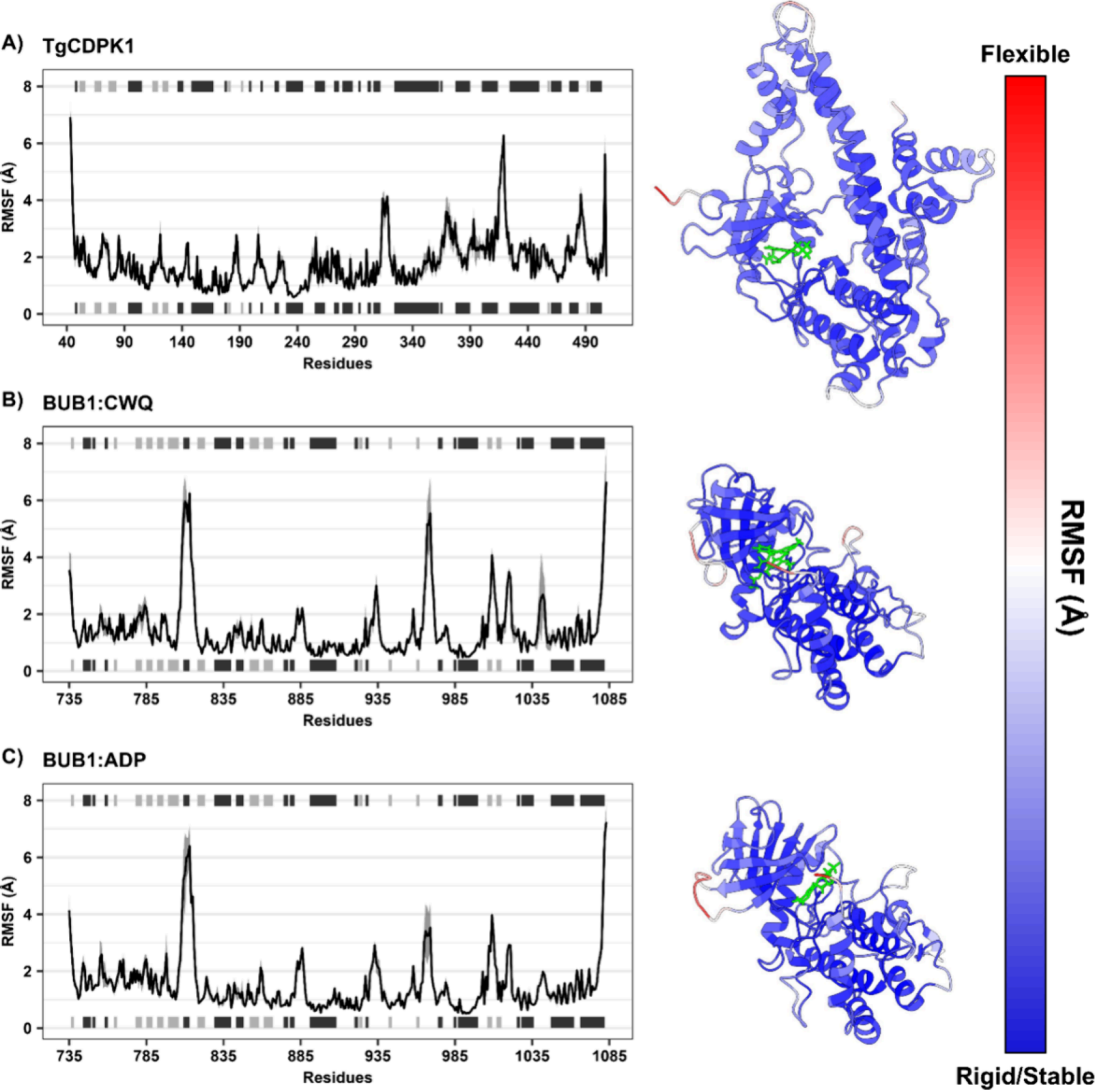
RMSF analysis of TgCDPK1 and BUB1 complexes: (A) TgCDPK1 complexed with UW2, (B) BUB1 complexed with CWQ, and (C) BUB1 complexed with ADP (with phosphorylated Ser969). Bar plots show atomic flexibility with secondary structure elements above (black: α-helices, gray: β-sheets). Protein structures colored by flexibility from blue (stable) to red (flexible), with ligands in green.

The RMSF profile of BUB1 complexed with CWQ revealed a globally stable conformation with low atomic fluctuations across most of the catalytic domain (Figure 4B). The active site region (residues 870–950) remained particularly rigid, with RMSF values consistently below 1.5 Å, indicating that CWQ binding preserves the structural integrity of the binding pocket. However, a prominent peak in flexibility was observed around residues 810–820, where RMSF values reached approximately 6.0 Å. This region likely corresponds to a solvent-exposed loop with high conformational mobility. Elevated fluctuations were also detected near residue 1000 and at the C-terminal end, a region commonly associated with intrinsic flexibility. Notably, the absence of phosphorylation at residue 969 in this system is associated with increased local flexibility, which may reflect the inactive or preactivation state of BUB1 prior to autophosphorylation.

The ADP-bound form of BUB1 displayed a similar overall RMSF pattern, with low fluctuations in the catalytic core (residues 870–950), reflecting a well-stabilized nucleotide-bound state (Figure 4C). Compared to the CWQ complex, the region around residues 810–820 showed slightly reduced flexibility, with RMSF values peaking at approximately 5.5 Å, suggesting a less dynamic loop conformation in the absence of phosphorylation. Minor peaks were also observed near residue 1000 and at the C-terminal tail, consistent with dynamic peripheral regions not directly involved in ligand binding. Importantly, the presence of a phosphorylated serine at position 969 (SEP969) correlates with decreased local flexibility, supporting its role in stabilizing the active conformation of BUB1 during the autophosphorylation-driven activation process.

To complete our dynamic analyses, detailed hydrogen bonding pattern analysis revealed distinct interaction profiles for each complex, with some key residues maintaining highly persistent contacts throughout the simulations (Figure S3). The TgCDPK1:UW2 complex displayed a focused hydrogen bond network centered on Tyr131, which formed a highly persistent interaction with the inhibitor, maintaining a 94.3% occupancy over the course of the simulation. This key anchoring interaction was complemented by two additional hydrogen bonds involving the carboxyl oxygen of Glu129 and the N5 amine group of UW2. These interactions occurred in two distinct configurations, in which the carboxylate oxygen of Glu129 alternately formed hydrogen bonds with the HN51 and HN52 hydrogen atoms of UW2’s N5 amine group (Figure S4). Both configurations occurred with similar frequency, exhibiting occupancy rates of 49.5 and 49%, respectively, indicating a dynamic yet consistent interaction that reinforces ligand stabilization.

The BUB1 complexes exhibited distinct but well-defined hydrogen bonding profiles (Figure S5). In the BUB1:CWQ complex, the most persistent interaction was observed between CWQ and Tyr869, with a remarkably high occupancy of 97.3%, suggesting a key anchoring role for this residue. Additional stable hydrogen bonds were formed with Asp946 and Met850, both presenting similar occupancy levels around 69%. A less frequent, yet structurally meaningful set of interactions was identified with Lys821, which engaged CWQ through two separate hydrogen atoms of its terminal amino group, each showing occupancies of approximately 30%.

In the BUB1:ADP complex, Tyr869 again formed a highly stable interaction with the ligand, reaching 96.3% occupancy (Figure S6). Two additional hydrogen bonds were observed between the carboxylate oxygen of Glu867 and the N3 amine group of ADP. These interactions alternated between two configurations involving different hydrogen atoms (HN31 and HN32), with occupancy rates of 49.2 and 47.9%, respectively, suggesting dynamic but sustained engagement.

The conservation of strong tyrosine-mediated hydrogen bonding across all complexes underscores its fundamental role in ligand recognition, with Tyr869 in BUB1 and Tyr131 in TgCDPK1 consistently exhibiting high occupancy and persistence throughout the simulations. However, each complex displays a distinct secondary interaction profile that may underlie differences in the ligand specificity and stability. The BUB1:CWQ complex presents the most extensive hydrogen bond network, involving not only Tyr869 but also Asp946, Met850, and Lys821, although the latter displays a more intermittent interaction pattern. In contrast, the BUB1:ADP complex maintains a similarly strong anchoring via Tyr869, but engages in fewer additional hydrogen bonds, primarily with Glu867 through two dynamic yet persistent configurations. The TgCDPK1:UW2 complex, while exhibiting fewer overall interactions, displays a highly stable binding mode characterized by continuous hydrogen bonding with Tyr131 and alternating, yet sustained, interactions with Glu129. These distinct patterns suggest that while tyrosine-mediated interactions serve as a common structural anchor, the surrounding network of secondary hydrogen bonds contributes to the differential binding behavior and potential selectivity of each ligand across the two kinases.

To further characterize the dynamic behavior of the binding sites, comparative analyses of the pocket volumes throughout the trajectories revealed marked differences among the three complexes (Figure 5). BUB1 complexed with CWQ exhibited the smallest and most stable pocket with an average volume of approximately 875 Å^3^ and minimal fluctuations over time, indicating a compact and rigid binding environment. In contrast, TgCDPK1 complexed with UW2 presented a moderate average pocket volume (∼1000 Å^3^), but with notably higher fluctuations and a gradual expansion trend, suggesting a more flexible and adaptable binding site. The BUB1:ADP complex, however, showed a substantially larger and more open pocket, maintaining an average volume close to 1700 Å^3^, with an initial phase of expansion, followed by stabilization.

**Figure 5:**
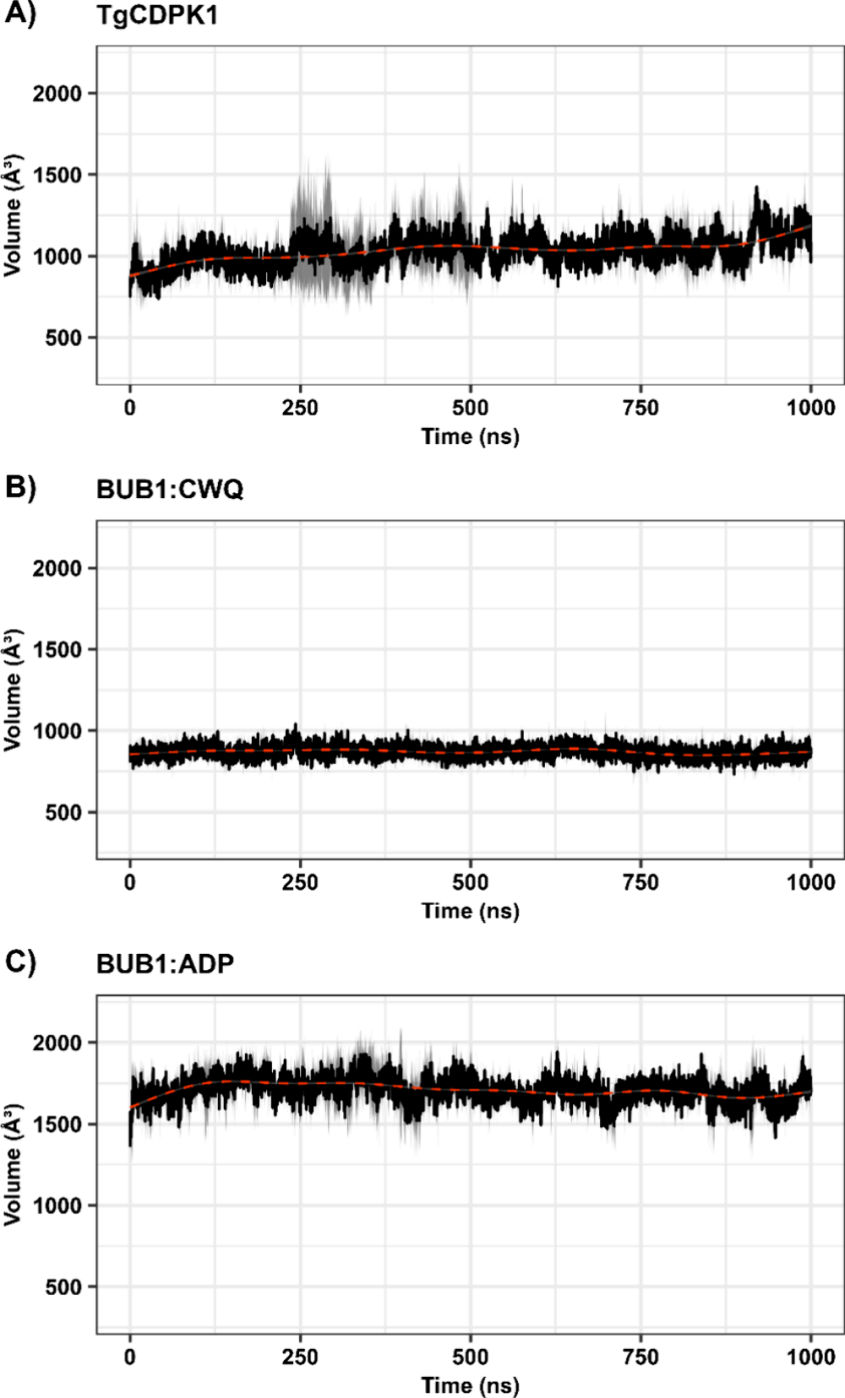
ATP-binding site volume dynamics during the MD simulations. Evolution of binding pocket volumes for (A) TgCDPK1 complexed with UW2, (B) BUB1 complexed with CWQ, and (C) BUB1 complexed with ADP. Black lines represent the average of independent replicates; gray areas indicate 95% confidence intervals. Red dashed lines denote trend lines.

To leverage these dynamic conformational changes for drug design, five representative structures were selected from each system based on clustering analysis of the ATP-binding site throughout the combined 2000 ns trajectory (Figure S7). K-means clustering was applied to residues within 5 Å of the bound ligand using RMSD as the similarity metric, allowing the identification of five distinct conformational clusters per complex. The centroid of each cluster was selected as a representative structure, ensuring the selection of statistically justified and structurally diverse conformations. This systematic approach captures the range of binding site plasticity observed during simulations, providing a robust ensemble for subsequent virtual screening.

This comprehensive dynamic analysis reveals that despite sharing a glycine gatekeeper, TgCDPK1 and BUB1 exhibit distinct binding site volumes depending on their bound ligands. BUB1 complexed with CWQ showed the most stable and compact pocket, TgCDPK1 complexed with UW2 presented an intermediate volume with higher conformational flexibility, and BUB1 complexed with ADP maintained the largest and most accessible binding site. These distinct volumetric profiles may offer crucial opportunities for selective inhibitor design. These findings set the stage for our subsequent virtual screening analyses, aimed at exploiting these conformational differences.

### Comparative Virtual Screening of Apicomplexan Kinase Inhibitors against TgCDPK1 and BUB1

To evaluate the potential selectivity of known apicomplexan kinase inhibitors between TgCDPK1 and BUB1, we chose seven distinct compounds: ATP (as reference ligand), BKI-1294, BKI-1517, BKI-1748, UW2, compound 25, and compound 32. These compounds were chosen based on their documented efficacy against apicomplexan kinases in previous studies,^17,32–37^ particularly their ability to inhibit CDPK1 orthologs across different parasitic species.

Before performing virtual screening, we conducted a structural similarity analysis among the selected compounds to assess potential correlations between the chemical structure and binding behavior. This step was essential to understanding how specific structural features might influence interactions of the ligand with TgCDPK1 and BUB1. Compounds with high chemical similarity were expected to exhibit comparable binding patterns and potentially similar selectivity profiles, whereas structurally distinct molecules might interact differently with the two kinases, despite targeting the same binding site.

To quantitatively assess these structural relationships, we employed the Tanimoto coefficient, which is a widely used metric for molecular similarity. This analysis provided insights into whether selectivity is primarily driven by specific chemical scaffolds or transcends distinct structural classes, thereby forming future strategies for designing selective inhibitors. Understanding these correlations before docking experiments allowed us to contextualize binding predictions and refine our interpretation of ligand-kinase interactions.

Results from this analysis confirmed that the selected compounds exhibited varying degrees of chemical similarity, which could influence their differential binding behavior. Initial structural similarity analyses among these ATP-competitive compounds revealed distinct correlation patterns, as demonstrated by hierarchical clustering and Tanimoto coefficient calculations (Figure 6A). The correlation matrix presented as a heatmap with hierarchical clustering showed significant variations in structural similarities, with coefficients ranging from 0.46 to 0.95. The dendrogram reveals three main clusters with ATP forming an isolated branch. Compound 32 and UW2 exhibited the highest correlation (Tanimoto coefficient of 0.95), attributed to their shared imidazopyridine core structure and similar cyclic substituents (cyclopropyl and cyclopropoxy groups). Another significant correlation was observed between BKI-1517 and BKI-1748 (Tanimoto coefficient = 0.87), both featuring a central pyrazole ring system with amide substituents.

**Figure 6:**
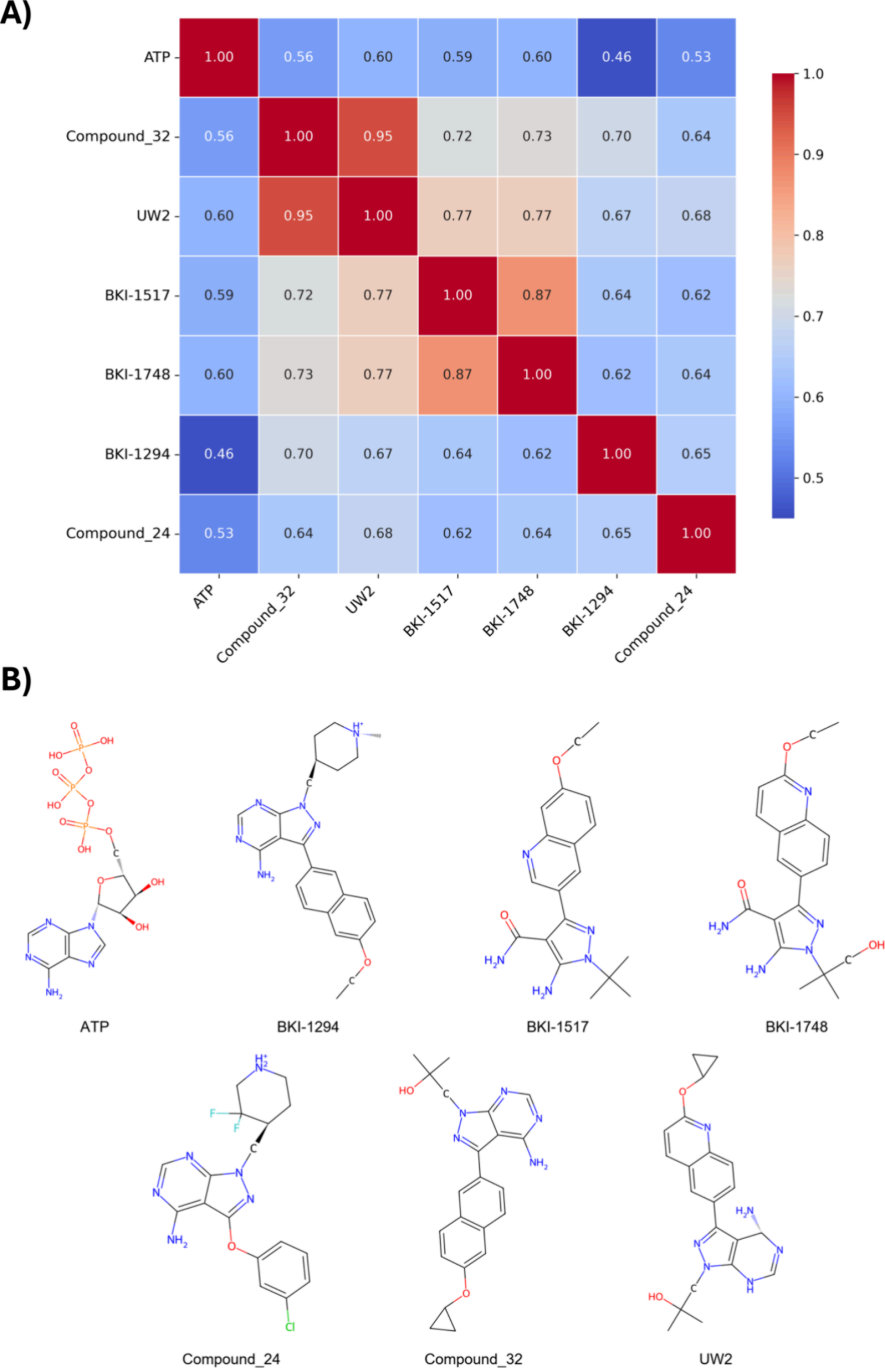
Analysis of ATP and apicomplexan kinase inhibitors. (A) Hierarchical clustering matrix showing Tanimoto similarity coefficients (blue = low to red = high similarity). (B) Chemical structures of ATP (reference) and six inhibitors used in virtual screening sharing common pharmacophoric features.

The structural characterization revealed several conserved features across these compounds (Figure 6B). As shown in the chemical structures, a fused heterocyclic core system is present in all synthetic compounds, while an amino group (NH2) is conserved across all molecules, including ATP. The compounds also contain a purine recognition region, analogous to ATP’s adenine moiety, and a hydrogen bond acceptor region implemented through N, O, or NH2 groups. Notably, ATP showed consistently lower correlations with all synthetic compounds (Tanimoto coefficients ranging from 0.46 to 0.60), which is expected given its distinct nucleotide structure with phosphate groups. The lowest correlation was observed between ATP and BKI-1294 (0.46), highlighting the structural divergence of this synthetic compound from the natural substrate, as evidenced by their distinct core structures shown in Figure 6B.

To validate the docking protocol, a docking validation was performed using five representative frames obtained from MD simulations of TgCDPK1 complexed with UW2, BUB1 complexed with ADP, and BUB1 complexed with CWQ (Figure 7). Only frames with Ligand-RMSD values ≤ 2.0 Å were selected for subsequent virtual screening. For the TgCDPK1 complexed with UW2, frames 1, 3, and 5 were retained (ligand-RMSD = 0.90, 0.94, and 0.72 Å, respectively), while frames 2 and 4 were excluded. In the BUB1 complexed with CWQ, all five frames showed excellent agreement with the reference pose (ligand-RMSD between 0.41 and 0.54 Å) and were included. For the BUB1 complexed with ADP, only frames 4 and 5 were selected (ligand-RMSD = 2.30 and 1.94 Å); although frame 4 slightly exceeded the cutoff, it was retained due to the influence of a flexible region in the ligand that affected the ligand-RMSD without compromising the overall binding mode.

**Figure 7:**
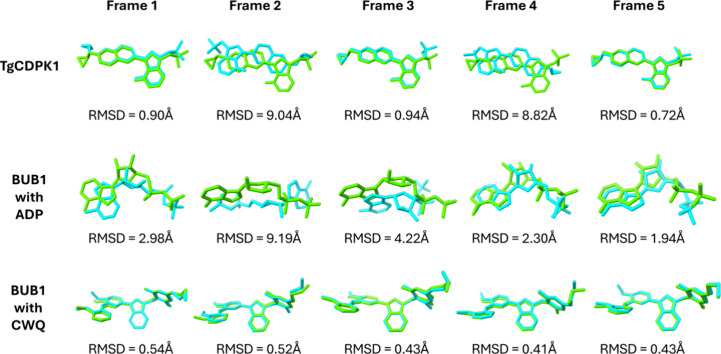
Docking validation of MD-derived conformations. Overlaid docked poses (cyan) are overlaid with original MD poses (green) for TgCDPK1 complexed with UW2, BUB1 complexed with ADP, and BUB1 complexed with CWQ.

Following protocol validation, we performed a virtual screening of the selected compounds against both TgCDPK1 and BUB1 using multiple conformational frames derived from molecular dynamics simulations. In TgCDPK1, inhibitors such as BKI-1294, Compound 32, and UW2 consistently yielded more favorable grid scores than ATP across all frames (Table 3). The differences were often substantial, with BKI-1294 outperforming ATP by up to 5 kcal/mol, and Compound 32 and UW2 also maintaining lower scores in most cases. In contrast, the behavior in BUB1 was more variable. ATP remained the top-scoring ligand in the majority of conformations, especially in frames derived from the ADP-bound state. In the CWQ-bound models, BKI-1294 surpassed ATP in Frames 2 and 3 by margins of 4.0 and 2.6 kcal/mol, respectively, although other inhibitors did not replicate this pattern. In several BUB1 frames, the synthetic compounds scored close to or above ATP, indicating a reduced binding affinity. Altogether, these results highlight distinct patterns of ligand accommodation across the two kinases, with a more consistent advantage observed for the inhibitors in TgCDPK1.

**Table 3:**
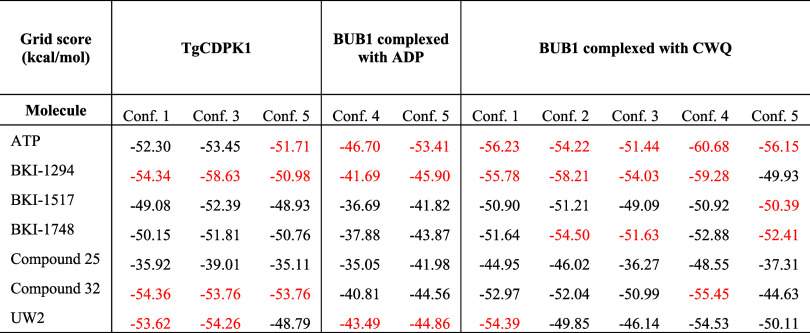
Grid Scores (kcal/mol) from Virtual Screening of ATP and Apicomplexan Kinase Inhibitors against TgCDPK1 and BUB1^a^

a Conf. (1–5) represents distinct protein conformations extracted from molecular dynamics trajectories. More negative grid score values indicate more favorable predicted binding energies. ATP serves as a reference ligand. Data are organized by receptor–ligand systems: TgCDPK1 alone, BUB1 complexed with ADP, and BUB1 complexed with CWQ inhibitor. The top-3 scoring molecules for each conformation are highlighted in red.

To elucidate the molecular basis underlying the distinct binding preferences observed in the virtual screening, we performed a comparative analysis of protein–ligand interactions across the three systems: TgCDPK1 complexed with UW2, BUB1 complexed with ADP, and BUB1 complexed with CWQ (Figure 8). Despite structural similarities between the kinases, the results revealed marked differences in interaction patterns and binding behavior.

**Figure 8:**
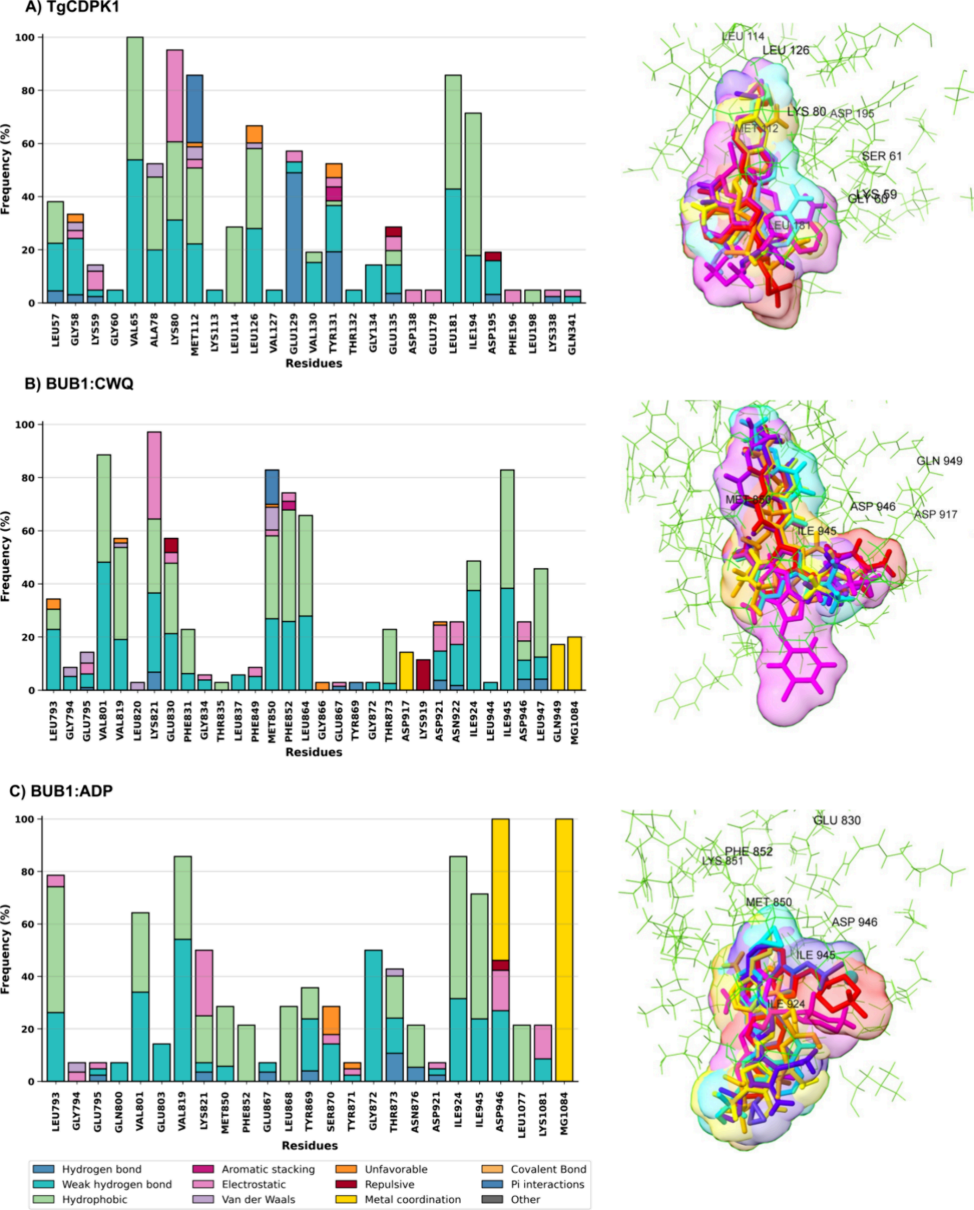
Ligand interaction profiles and binding modes in TgCDPK1 and BUB1. Stacked bar plots (left) and Surface representations (right) illustrate the ligand binding modes within each pocket and illustrate the interaction profiles for (A) TgCDPK1 complexed with UW2, (B) BUB1 complexed with CWQ, and (C) BUB1 complexed with ADP.

TgCDPK1 exhibited a dense and focused interaction network with ligands consistently engaging a well-defined subset of residues through multiple interaction types. Key contacts were concentrated around Val65, Ala78, Lys80, Met112, Glu129, Tyr131, Leu181, and Ile194. These residues were contributed through a combination of hydrophobic contacts, weak hydrogen bonds, and electrostatic interactions, frequently forming overlapping interaction types. This highly localized and layered interaction pattern reflects a stable and optimized binding mode, with ligands adopting a consistently organized spatial arrangement within the binding pocket. These characteristics were mirrored in the virtual screening results in which ATP was consistently outperformed by multiple inhibitors across all frames, particularly BKI-1294 and Compound 32.

In contrast, BUB1 complexed with ADP displayed a more dispersed and structurally distinct interaction profile. Although this system showed the highest total interaction frequency, the density was strongly influenced by metal coordination involving the phosphate groups of ATP and the Mg^2+^ ion, particularly through Asp946 and MG1084 (Figure S8). Additional residues such as Val801, Lys821, Met850, Gly872, Ile925, and Ile945 were frequently involved, engaging ligands via hydrophobic and electrostatic interactions. The overall network, however, was broader and less focused, with ligands interacting across a wider range of residues and displaying less spatial organization.

BUB1 complexed with CWQ presented an intermediate interaction profile. While not as dense as those in TgCDPK1, several residues demonstrated consistent ligand engagement, including Val801, Lys821, Met850, Phe852, Glu867, Ile925, Ile945, and Asp946. These interactions were distributed across different types: hydrophobic, hydrogen bonds, electrostatic, and metal coordination, suggesting a more flexible and variable binding pocket. This is consistent with the virtual screening results, in which some inhibitors showed favorable scores in specific frames but failed to consistently outperform ATP. Ligands in this system also displayed greater variability in positioning within the pocket, further suggesting a less well-defined binding mode.

Together, these findings highlight key mechanistic distinctions. TgCDPK1 is characterized by a compact and interaction-rich binding site, where a small set of residues establishes multiple contact types that stabilize ligands in a conserved and organized pose. In contrast, BUB1 complexes, particularly with ADP, exhibit interaction networks that are either overly dispersed or shaped by features specific to ATP coordination, such as metal-mediated contacts. These differences are directly reflected in the docking outcomes: inhibitors consistently outperformed ATP in TgCDPK1, but not in BUB1. This provides a structural rationale for the observed selectivity, supporting TgCDPK1 as a promising target for the development of parasite-selective kinase inhibitors.

## Discussion

In this study, we identified and characterized the human mitotic checkpoint serine/threonine-protein kinase BUB1 as possessing a glycine residue at the gatekeeper position, a feature previously considered unique to apicomplexan kinases such as TgCDPK1. This computational observation suggests that the presence of a glycine residue at the gatekeeper position may not be exclusive to parasite kinases, as previously considered, which may have implications for the development of selective inhibitors targeting TgCDPK1.

To contextualize our findings, it is critical to understand the functional differences between TgCDPK1 and BUB1, despite their shared glycine gatekeeper feature. TgCDPK1 functions as a calcium-dependent kinase in *T. gondii*, regulating essential processes such as host cell invasion, egress, and motility.^6–9^ Its activation mechanism begins with Ca^2+^ binding to EF-hand domains, triggering a substantial conformational change that relieves autoinhibition and exposes the catalytic site.^10,11^ This calcium-dependent regulation is crucial for the parasite’s life cycle transitions and represents a specialized adaptation in apicomplexan organisms.^2,3^ The unique structural aspects of TgCDPK1 have been previously characterized.^10–12^ BUB1, conversely, is a mitotic checkpoint kinase in humans that monitors chromosome alignment during cell division.^24^ Its activity is primarily regulated by autophosphorylation at Ser969 in the P+1 loop (not present on TgCDPK1), which removes steric hindrance from the active site.^28,29^ BUB1 is widely expressed in various human tissues, including the thymus, colon, spleen, and lung,^25^ where it plays critical roles in ensuring accurate chromosome segregation. Its detailed structural characterization has been previously reported.^39^

While both kinases share structurally similar catalytic domains with glycine gatekeepers (Gly128 in TgCDPK1 and Gly937 in BUB1), they exhibit distinct interaction patterns and binding site properties. Notably, BUB1 utilizes a unique metal coordination network involving Mg^2+^ ions and Asp946 that is absent in TgCDPK1, contributing to a more distributed interaction pattern compared to the focused interactions observed in TgCDPK1. This difference in coordination chemistry likely influences how each kinase recognizes and processes substrates and inhibitors. Additionally, their overall domain organization differs significantly - TgCDPK1 contains four C-terminal EF-hand motifs forming a calcium-sensing apparatus unique to apicomplexan parasites,^12^ while BUB1 features N-terminal TPR domains and interactions with other checkpoint proteins like Bub3.^39^

The glycine gatekeeper residue in TgCDPK1 (Gly128) has been a cornerstone for the development of selective inhibitors, primarily because it creates an enlarged hydrophobic pocket adjacent to the ATP-binding site, which is absent in most human kinases.^10,11^ This structural uniqueness has been exploited to design bumped kinase inhibitors (BKIs) that selectively target TgCDPK1 without affecting human kinases, thereby minimizing potential off-target effects.^12,16^ However, our identification of BUB1 as a human kinase with a glycine gatekeeper calls for a re-evaluation of this strategy.

Despite the shared glycine gatekeeper, our comprehensive structural analyses revealed critical differences between TgCDPK1 and BUB1 that can be leveraged for selective inhibitor design. Notably, the ATP-binding pockets of both kinases exhibit similar volumes and depths but differ significantly in their electrostatic surface potentials and residue composition. TgCDPK1 displays a mixed electrostatic potential with both positive and negative regions, whereas BUB1 exhibits a predominantly negative potential. These differences align with previous studies emphasizing that factors beyond the gatekeeper residue, such as the overall shape, charge distribution, and hydrophobicity of the binding pocket, play crucial roles in determining inhibitor selectivity.^13,14^

Our molecular dynamics simulations further elucidated the dynamic behavior of the ligand-binding sites in both kinases. The stable binding mode of UW2 in TgCDPK1, characterized by strong interactions with key residues such as Tyr131 and Glu129, underscores the potential for designing inhibitors that take advantage of these specific interactions.^38^ In contrast, BUB1 complexes exhibited more dispersed and less interaction-dense binding profiles with ligands engaging a broader set of residues in a less organized manner, which may underlie their weaker and less consistent binding affinities for the same inhibitors.

The virtual screening results corroborated these findings, as known apicomplexan kinase inhibitors displayed consistently higher and more stable affinity for TgCDPK1 compared to BUB1. Despite the presence of a conserved glycine gatekeeper in both kinases, the inhibitors exhibited more favorable and organized binding modes in TgCDPK1, characterized by denser and more focused interaction networks. These results suggest that factors beyond the gatekeeper residue, such as binding site organization, interaction density, and pocket architecture, are critical for achieving selective inhibition.^13,14^

Our findings align with previous studies emphasizing the importance of the entire binding site environment in achieving selectivity. Liu and Gray^13^ highlighted that inhibitor design should consider not just the gatekeeper residue but also other determinants of specificity within the kinase domain. Zuccotto et al.^14^ further demonstrated that subtle differences in active site conformation can be exploited for selective inhibition.

The discovery of a human kinase with a glycine gatekeeper also underscores the necessity for thorough structural and functional analyses in target validation. While the glycine gatekeeper in TgCDPK1 has been a focal point for drug development, our results indicate that relying solely on this feature may not suffice to avoid off-target effects on human kinases like BUB1. Given the crucial role of BUB1 in mitotic checkpoint regulation,^24^ its unintended inhibition could have significant detrimental clinical consequences.

Therefore, our study suggests that future inhibitor design efforts should incorporate a holistic approach that accounts for the entire ATP-binding pocket, including residue composition, electrostatic properties, and dynamic behavior. By focusing on the unique structural and dynamic features of TgCDPK1’s binding site, it is possible to design inhibitors that achieve high selectivity and efficacy while minimizing potential off-target effects on human kinases.

Limitations of our study include the reliance on computational simulations, which, while powerful, require experimental validation. Future work should involve biochemical and cellular assays to test the selectivity and efficacy of proposed inhibitors against both TgCDPK1 and BUB1. Additionally, exploring the structural diversity of human kinases may reveal other kinases with similar features, requiring an even more refined approach to selective inhibitor design.

In conclusion, our comprehensive analysis deepens our understanding of the structural basis for the selective inhibition of TgCDPK1. The identification of BUB1 as a human kinase with a glycine gatekeeper challenges previous assumptions but also provides an opportunity to enhance inhibitor selectivity by exploiting differences in the binding pocket environment. Our findings pave the way for the rational design of next-generation TgCDPK1 inhibitors with improved selectivity and efficacy, contributing to the development of safer and more effective therapeutics against toxoplasmosis.

## Methods

The comprehensive computational methodology employed in this investigation is summarized in Figure 9, which illustrates the systematic workflow encompassing sequence and structure-based similarity searches, comparative binding site analyses, molecular dynamics simulations, and virtual screening protocols. This integrated approach enabled the identification and characterization of human kinases sharing structural features with TgCDPK1, followed by a detailed evaluation of their potential for selective inhibitor design.

**Figure 9:**
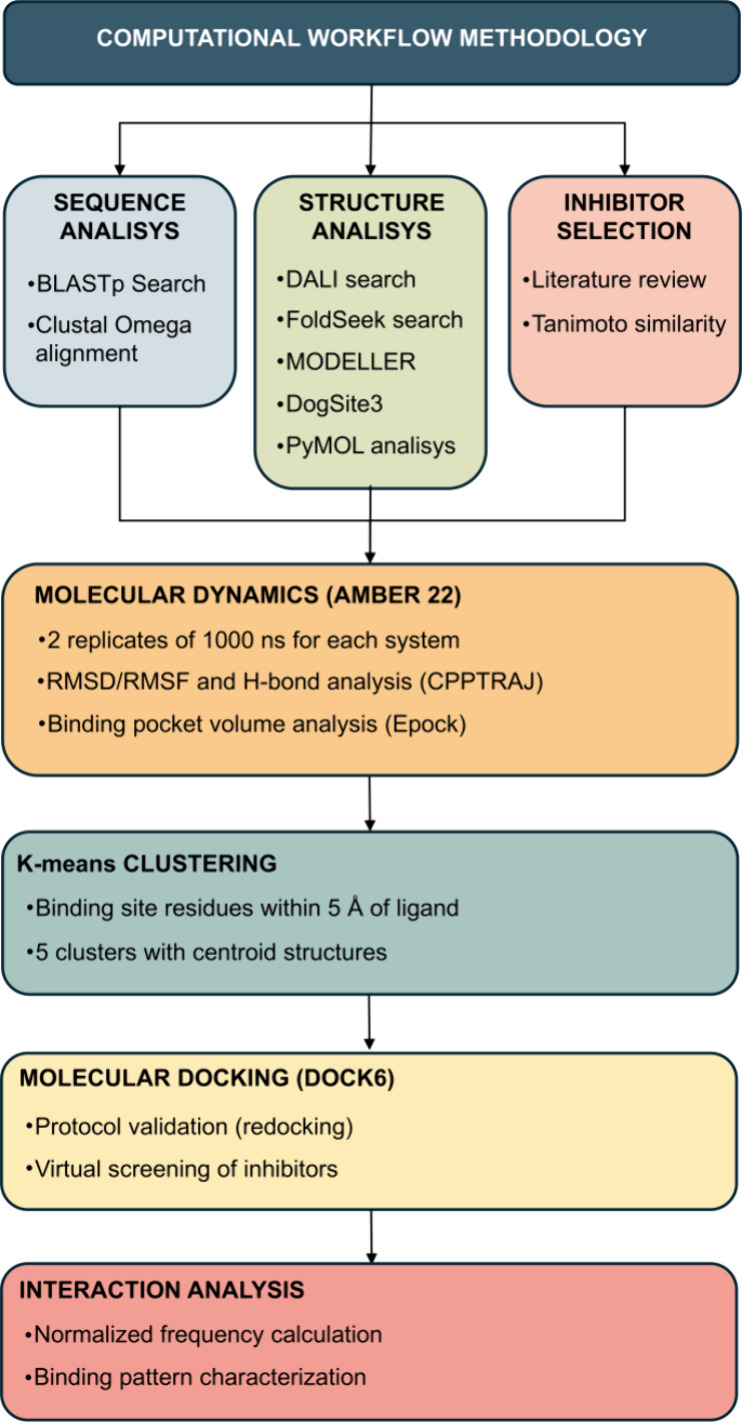
Workflow of the methodology.

### Protein Structure Preparation and Model Generation

Crystallographic structures of TgCDPK1 (3I7B) and BUB1 (6F7B) were obtained from the Protein Data Bank for structural analysis. Additional protein–ligand complex structures were retrieved for molecular dynamics and virtual screening investigations, including TgCDPK1 complexed with UW2 (4TZR), BUB1 complexed with ADP (5DMZ), and BUB1 complexed with CWQ (6F7B). Structural data for human kinase domains were sourced from experimentally determined structures in the Protein Data Bank for CHK2 (2YCF), KCC1A (4FG9), KCC1D (6T29), KS6A5 N-Terminal (1VZO), and MARK1 (6C9D) and computationally predicted models from the AlphaFold database for KCC1G, KPSH1, KPSH2, MYLK4, KS6A5 C-terminal, and SIK2.

Missing residues were reconstructed using MODELER^40^ via the ChimeraX interface.^41^ Models were selected based on the lowest discrete optimized protein energy (DOPE) scores.^42^ The N-terminal kinase domain of KS6A5 required additional comparative modeling using crystallographic structures 1VZO and 4FG9 as templates, which provided optimal sequence coverage and high-resolution structural data.

Sequence alignment was performed with Clustal Omega^43^ using default parameters for protein sequences, then converted to the PIR format for MODELER compatibility. Models were generated using MODELER’s ‘automodel’ class with ‘slow’ refinement mode and conjugate gradients optimization (maximum 300 iterations). One hundred independent models were produced to ensure thorough conformational sampling.

Model quality was assessed by using multiple validation metrics. Initial selection was based on DOPE scores, which evaluate protein structure statistical potentials. Selected models underwent further validation through the Swiss-Model platform,^44^ including MolProbity analysis^45^ and Ramachandran plot evaluation.^46^

### Structural and Sequence Analysis of Protein Kinases

The sequence-based analysis utilized the Basic Local Alignment Search Tool for proteins (BLASTp) algorithm^47^ with TgCDPK1’s amino acid sequence (UniProtKB: Q9BJF5) as the query against the comprehensive human proteome database from the UniProt database.^48^ The search parameters were rigorously defined to ensure high-confidence matches: sequence identity threshold of 40%, alignment coverage exceeding 40%, *E*-value cutoff below 1 × 10^–10^, and alignment scores greater than 120. Initial results were subjected to additional filtering to ensure complete coverage of TgCDPK1’s catalytic kinase domain (residues 51–308), yielding ten human protein sequences meeting all criteria.

Parallel structure-based analysis was implemented using the DALI server and FoldSeek,^23^ employing TgCDPK1’s crystallographic structure (3I7B) as the search template. For the DALI server, this investigation encompassed two distinct structural databases: the Protein Data Bank (PDB), containing experimentally determined structures, and the AlphaFold database of computationally predicted protein models.^49^ The FoldSeek analysis included searches against multiple databases: pdb100, af-db proteome, af-db50, and af-db SwissProt. The structural search specifically focused on identifying human kinases possessing a glycine residue at the gatekeeper position, which is a critical structural feature of TgCDPK1.

Detailed characterization of ATP-binding sites was performed using the DogSite3 algorithm through the Protein Plus platform.^50^ This analysis generated quantitative parameters, including pocket volumes and depths, enabling systematic comparison across structures. Binding site architecture was further evaluated through the identification and analysis of pocket-lining residues. Structural alignments performed in PyMOL^51^ facilitated direct comparison of binding site topology between TgCDPK1 and identified human kinases.

Electrostatic properties of the binding sites were characterized through a multistep computational protocol. Initial structure preparation was conducted using the PDB 2PQR^52^ server at physiological pH (7.4), employing the PARSE force field^53^ parameters. Protonation states were assigned using PROPKA,^54^ followed by calculation of electrostatic potential surfaces via the APBS Electrostatics plugin^55^ in PyMOL. For these calculations, dielectric constants were adjusted to account for solvent effects, with a protein dielectric constant of 2.0 and a solvent dielectric constant of 78.5, following standard parameters for aqueous environments. This analysis concentrated specifically on ATP-binding pocket regions, providing insights into the local electrostatic environments that influence ligand recognition and binding specificity. The comprehensive characterization of binding site properties established a foundation for understanding the molecular basis of the selective inhibitor design.

### Molecular Dynamics Simulations

Molecular dynamics simulations were conducted on three distinct protein–ligand systems (TgCDPK1 complexed with UW2, BUB1 complexed with ADP, and BUB1 complexed with CWQ) using AMBER 22.^56^ The molecular interactions within protein domains were characterized using the Amber ff19SB force field,^57^ while ligand parametrization was accomplished through the Generalized Amber Force Field (GAFF)^58^ utilizing AM1-BCC charge calculations implemented in ANTECHAMBER.^59^ Long-range electrostatic interactions were treated using the Particle Mesh Ewald (PME) algorithm^60^ with a defined cutoff radius of 10 Å for short-range interactions.

Each system was constructed within a triclinic simulation box under periodic boundary conditions, maintaining a minimum distance of 10 Å between the protein atoms and box boundaries. Solvation was achieved using the OPC water model,^61^ with system neutralization accomplished through the addition of Cl^1–^ and Na^1+^ counterions. Energy minimization proceeded through a two-stage protocol: initial minimization comprised 2000 steps (1000 steepest descent followed by 1000 conjugate-gradient) with harmonic position restraints (force constant: 5 kcal mol^–1^ Å^–2^) applied to heavy atoms, followed by unrestrained minimization for 5000 steps (2500 steepest descent and 2500 conjugate-gradient).

Initial atomic velocities were assigned according to the Maxwell–Boltzmann distribution corresponding to a temperature of 20 K. Temperature elevation to 300 K was accomplished through a carefully controlled 1 ns heating protocol utilizing the Langevin thermostat,^62^ with harmonic position restraints (force constant: 10 kcal mol^–1^ Å^–2^) maintained on heavy atoms. The heating phase was followed by a systematic equilibration procedure comprising nine sequential 500 ps simulations, during which positional restraints were progressively reduced to zero. Production phase molecular dynamics were subsequently performed for 1 μs at 300 K in the isothermal–isobaric (NPT) ensemble,^63^ employing isotropic pressure coupling at 1 atm.

Multiple simulation conditions were implemented to investigate the effects of metal ion coordination and post-translational modifications on binding site dynamics. The BUB1 complexed with ADP was simulated with a single Mg^2+^ ion and Ser969 in its phosphorylated state, enabling investigation of the coupled effects of metal coordination and protein phosphorylation on pocket architecture. Conversely, the BUB1 complexed with CWQ was simulated with one Mg^2+^ ion present while maintaining Ser969 in its unphosphorylated form, providing a reference state for comparative analysis of phosphorylation-dependent conformational dynamics.

### Trajectory Analysis and Binding Site Characterization

Molecular dynamics trajectories were analyzed using the CPPTRAJ module^64^ within AMBER. Root mean square deviation (RMSD) and root-mean-square fluctuation (RMSF) calculations were performed on protein and ligand heavy atoms, using the initial production phase structures as reference configurations. Hydrogen bond interactions were quantitatively assessed using geometric criteria of 3.5 Å distance and 120° angle cutoffs, with occupancy values calculated as the percentage of trajectory frames meeting these defined parameters.

A k-means clustering analysis was implemented to obtain representative structures for virtual screening procedures. For each molecular system, two independent 1000 ns trajectories were concatenated to provide a comprehensive conformational ensemble spanning 2000 ns of simulation time. Implementation of the clustering protocol utilized the CPPTRAJ module of AMBER22. For all systems, structural alignment prior to clustering was performed exclusively on the basis of residues comprising the ATP-binding site, allowing a more focused evaluation of local conformational variability. The binding site residues selected for clustering analysis were defined as those within 5 Å of the respective ligands. The k-means algorithm was configured to generate five distinct clusters, with pairwise RMSD serving as the similarity metric. The centroid structure of each cluster, representing the conformation with minimal cumulative RMSD to all other members within the same cluster, was selected for subsequent virtual screening analyses. This approach ensured the selection of energetically favorable and structurally diverse protein conformations, thereby enhancing the robustness of the virtual screening procedure.

The temporal evolution of binding site volumes was characterized using the Epock program.^65^ For each system, a spherical region of interest was defined by using a 10 Å radius centered on the geometric center of the respective ligands. Volume calculations were performed considering only the available void space in the absence of ligands, providing a quantitative assessment of pocket accessibility throughout the trajectories.

### Identification and Selection of Inhibitors for Virtual Screening

A systematic literature survey was conducted through PubMed to identify previously characterized inhibitors of apicomplexan kinases. The search strategy employed specific terms targeting CDPK1 inhibitors across multiple apicomplexan species: Besnoitia besnoiti, Cryptosporidium parvum, Neospora caninum, and CDPK4 inhibitors of T. gondii. Based on documented antiparasitic activity and the availability of comprehensive structural information, seven compounds were selected for virtual screening analysis: ATP (serving as the canonical ligand reference), BKI-1294, BKI-1517, BKI-1748, UW2, compound 25, and compound 32.

Compounds were prepared for docking by using ChimeraX software. Preparation included the addition of hydrogen atoms at physiological pH (7.4) to establish appropriate protonation states for accurate representation of ligand properties during virtual screening.

Subsequent quantitative analysis of structural relationships among the selected compounds was performed by using Tanimoto similarity metrics. Molecular fingerprints were generated employing MACCS (Molecular ACCess System) keys,^67^ comprising 166 predefined structural descriptors. Molecular structure processing and MACCS fingerprint generation were executed using the RDKit cheminformatics library.^68^

The degree of structural similarity between compound pairs was quantified through the calculation of Tanimoto coefficients (Tc), including comparisons with ATP. These pairwise similarity measurements were consolidated into a symmetrical similarity matrix, providing a comprehensive framework for analyzing structural relationships within the compound set. To facilitate the interpretation of these molecular similarities and identify potential structural clustering patterns, the similarity matrix was visualized through a hierarchically clustered heatmap representation, generated using the seaborn visualization library in Python.^69^

### Molecular Docking Studies

Molecular docking was performed using DOCK6 (Version 6.10) with an Anchor-and-Grow algorithm and force field-based scoring.^70^ The docking protocol implemented an Anchor-and-Grow algorithm with force field-based scoring. Initially, a series of docking validations were performed to validate the protocol. Docking validation was performed using molecular-dynamics-derived reference poses of TgCDPK1 complexed with UW2, BUB1 complexed with ADP, and BUB1 complexed with CWQ rather than crystallographic structures. Ligands were extracted from their MD-optimized positions and redocked to assess the protocol’s ability to reproduce these conformations.

The binding site was defined utilizing SPHGEN and SPHERE_SELECTOR programs, with spheres mapping the binding site surface configured with minimum and maximum radii of 1.4 and 5.0 Å, respectively. The center of mass of each ligand was employed as the central point for the site definition. The docking box was automatically constructed using SHOWBOX, accounting for the binding cavity volume, and the energy grid for scoring calculations was generated using the GRID tool.

Frames exhibiting Ligand-RMSD values of less than 2.0 Å were selected for subsequent virtual screening. An exception was made for frame 2 of BUB1 complexed with ADP, which was included despite an RMSD of 2.06 Å due to its potential structural significance. Ligand-RMSD values were calculated by considering all heavy atoms of each compound, comparing docking validation poses with their corresponding MD-derived reference conformations.

The validated frames were subsequently employed for virtual screening of ATP and six known apicomplexan kinase inhibitors: BKI-1294, BKI-1517, BKI-1748, UW2, compound 25, and compound 32. The flexible ligand protocol was utilized with enhanced sampling parameters, specifically increasing the ‘pruning max orients’ command from the default 100–1000 to ensure comprehensive conformational exploration. Grid scores were calculated for each ligand-protein complex, with more negative values indicating more favorable predicted binding energies. For each ligand in each protein conformation, the pose with the lowest energy (most negative grid score) was used for subsequent analysis and comparison. This systematic approach enabled comparative analysis of binding preferences between TgCDPK1 and BUB1, with particular emphasis on the relative affinities of apicomplexan inhibitors versus ATP in each protein system.

A comprehensive interaction analysis was conducted to characterize the noncovalent binding patterns between TgCDPK1/BUB1 and the virtual screening compounds, comprising six apicomplexan kinase inhibitors (BKI-1294, BKI-1517, BKI-1748, UW2, compound 25, and compound 32) and ATP. The analysis encompassed multiple docking poses generated for each ligand across different protein conformational states derived from TgCDPK1 complexed with UW2, BUB1 complexed with ADP, and BUB1 complexed with CWQ systems. For each ligand in each protein conformation, the single pose with the most favorable (most negative) Grid Score was selected for analysis and comparison. The red-highlighted values in Table 3 indicate the three best-performing compounds within each protein conformation; the single pose with the most favorable (most negative) Grid Score was selected for analysis and comparison. The red-highlighted values in Table 3 indicate the three best-performing compounds within each protein conformation.

Interaction data were normalized across protein–ligand complexes to standardize comparisons. The normalization accounted for variations in the interaction frequencies among residues across different poses and ligands. The process quantified and analyzed poses per system and determined the residue participation frequency in various interaction types (hydrogen bonds, hydrophobic interactions, electrostatic interactions, van der Waals forces, aromatic stacking, metal coordination, and pi interactions).

The interaction frequencies were normalized according to the following equation:

$$\mathrm{normalized}\;\mathrm{frequency}(\%)=\left(S\cdot \frac{F}{T}\cdot 100\right)/N$$

where *S* represents the number of systems involving a residue, *F* denotes the frequency of a specific interaction type, *T* is the total number of residue interactions, and *N* represents the total number of analyzed systems. This normalization methodology preserved the relative significance of each interaction type while accounting for the inherent variability in interaction patterns across different protein–ligand complexes.

The resulting normalized data, expressed as percentages, established a standardized framework for comparative analysis of protein–ligand interactions across all virtual screening poses. This approach facilitated the systematic interpretation of binding patterns between TgCDPK1/BUB1 and the screened compounds, enabling the quantitative assessment of differential binding preferences and interaction specificities.

## Supplementary Material



## Notes

Electrostatic potential analysis of ATP-binding pockets for human kinases lacking the glycine gatekeeper residue (Figure S1); root mean square deviation (RMSD) analysis of protein and ligand trajectories during molecular dynamics simulations for TgCDPK1 and BUB1 complexes (Figure S2); detailed hydrogen bond interaction patterns and occupancy profiles for TgCDPK1 complexed with UW2, BUB1 complexed with CWQ, and BUB1 complexed with ADP throughout 1 μs simulations (Figure S3); visualization of specific hydrogen bond interactions in each complex (Figures S4–S6); cluster analysis of ATP-binding site conformations over 2000 ns molecular dynamics simulations (Figure S7); and coordination of ATP by Mg^2+^ and Asp946 in BUB1 (Figure S8) (PDF)
