# Supplementary material for: Computational Structural Comparison of Toxoplasma gondii CDPK1 and Human BUB1 kinases: Implications for Selective Inhibitor Design
Source: ACS Omega. 2025 Jun 11;10(24):25415–31. doi: 10.1021/acsomega.5c00640 (PMC12199047; doi:10.1021/acsomega.5c00640)
Supplement: Supplementary file 1 [file ao5c00640_si_001.pdf]

Supporting information for

Computational Structural Comparison of *Toxoplasma gondii* CDPK1 and Human BUB1 kinases: Implications for Selective Inhibitor Design

João Pedro Bezerra Carvalho[1,2], Deborah Antunes[1*], Daniel Adesse[2,3], Ana Carolina Guimarães[1]

[1]Laboratory for Applied Genomics and Bioinnovations, Oswaldo Cruz Institute (IOC - FIOCRUZ), Rio de Janeiro, Brazil
[2]Structural Biology laboratory, Oswaldo Cruz Institute (IOC - FIOCRUZ), Rio de Janeiro, Brazil
[3]Laboratory of Ocular Immunology and Transplantation, Bascom Palmer Eye Institute, Miller School of Medicine, University of Miami, Miami, Florida, USA.

(*) Corresponding Author: E-mail: deborah.santos@fiocruz.br

Contents

Figure S1. ATP-Binding Pocket of human kinases without glycine as a gatekeep residue. S2
Figure S2. RMSD Analysis Over Time for TgCDPK1 and BUB1 Proteins and Ligands in Different Complexes...S3
Figure S3. Hydrogen bond analysis of TgCDPK1 and BUB1 complexes with their respective ligands. S4
Figure S4. Hydrogen bond interactions in the TgCDPK1 complexed with UW2..............................S5
Figure S5. Hydrogen bond interactions in the BUB1 complexed with CWQ....................................S5
Figure S6. Hydrogen bond interactions in the BUB1 complexed with ADP S5
Figure S7. Cluster analysis of ATP-binding site conformations over 2000 ns MD simulations.........S6
Figure S8. Coordination of ATP by Mg^{2+} and Asp946 in BUB1 ....S7

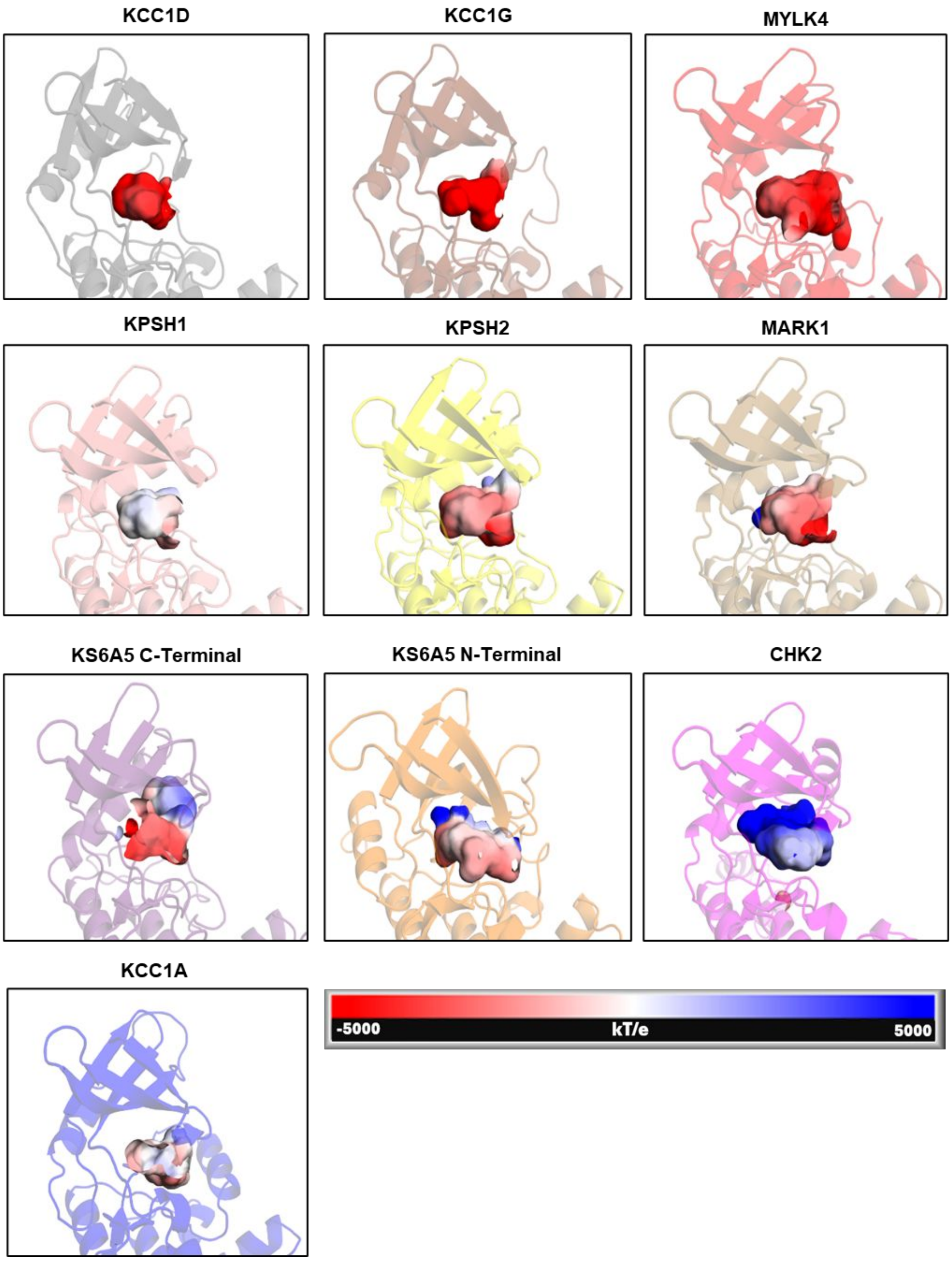

Figure S1. ATP-Binding Pocket of human kinases without glycine as a gatekeep residue. The surface potentials are shown for KCC1D, KCC1G, MYLK4, KPSH1, KPSH2, MARK1, KS6A5 (C-terminal and N-terminal domains), CHK2, and KCC1A. The electrostatic potential is displayed using a color gradient from red (negative, -5000 kT/e) to blue (positive, +5000 kT/e), as indicated by the scale bar. The visualization highlights the distinct electrostatic characteristics of the ATP-binding cavities among these kinases.

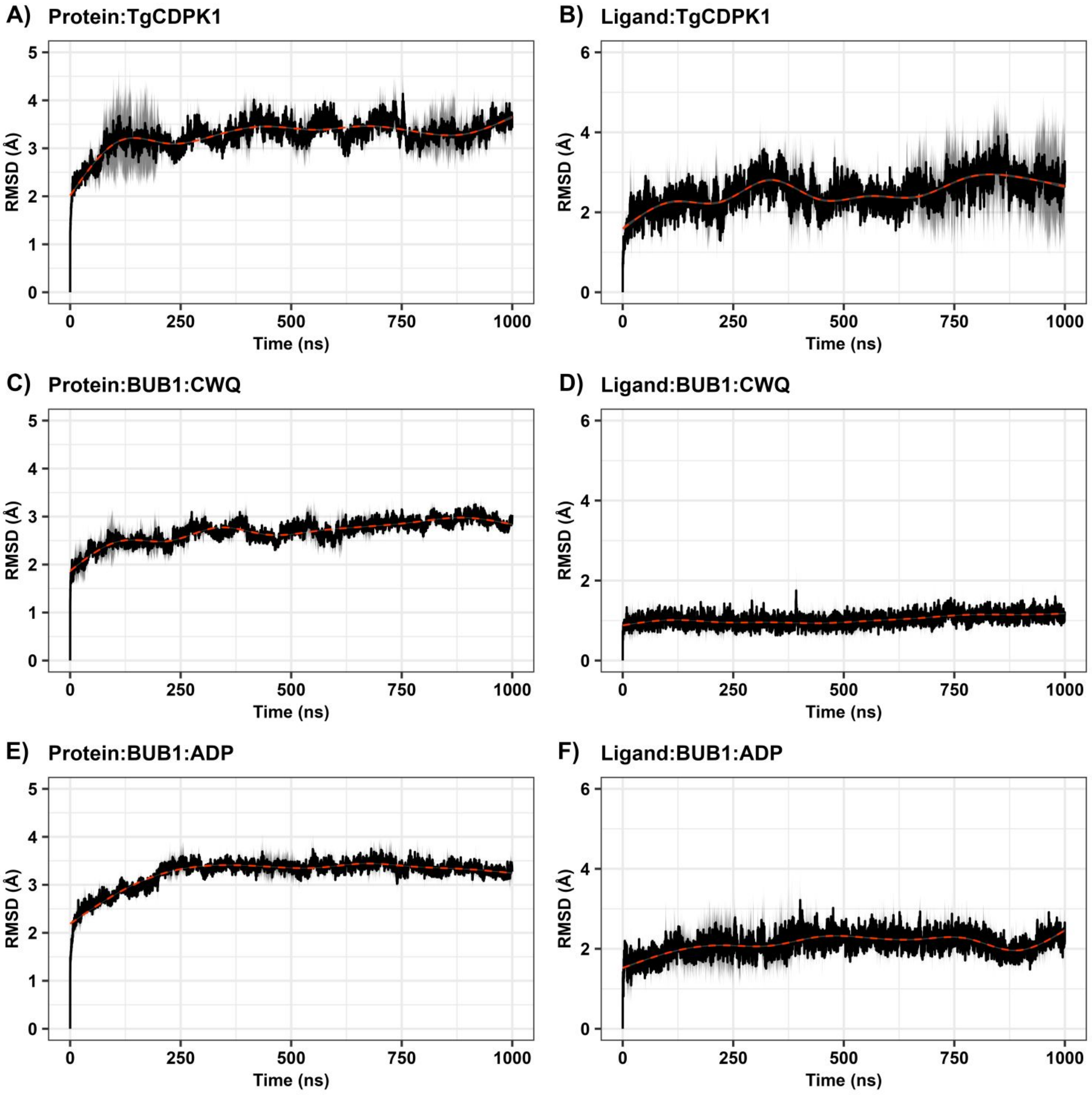

Figure S2. RMSD Analysis Over Time for TgCDPK1 and BUB1 Proteins and Ligands in Different Complexes. Time evolution of RMSD values for protein (left panels) and ligand heavy atoms (right panels) relative to their initial conformations at the start of production dynamics. (A-B) TgCDPK1:UW2 protein and ligand fluctuations. (C-D) BUB1:CWQ complex protein and ligand fluctuations. (E-F) BUB1:ADP complex protein and ligand fluctuations. Black lines represent the average of independent replicates; gray areas indicate 95% confidence intervals. Red dashed lines denote trend lines.

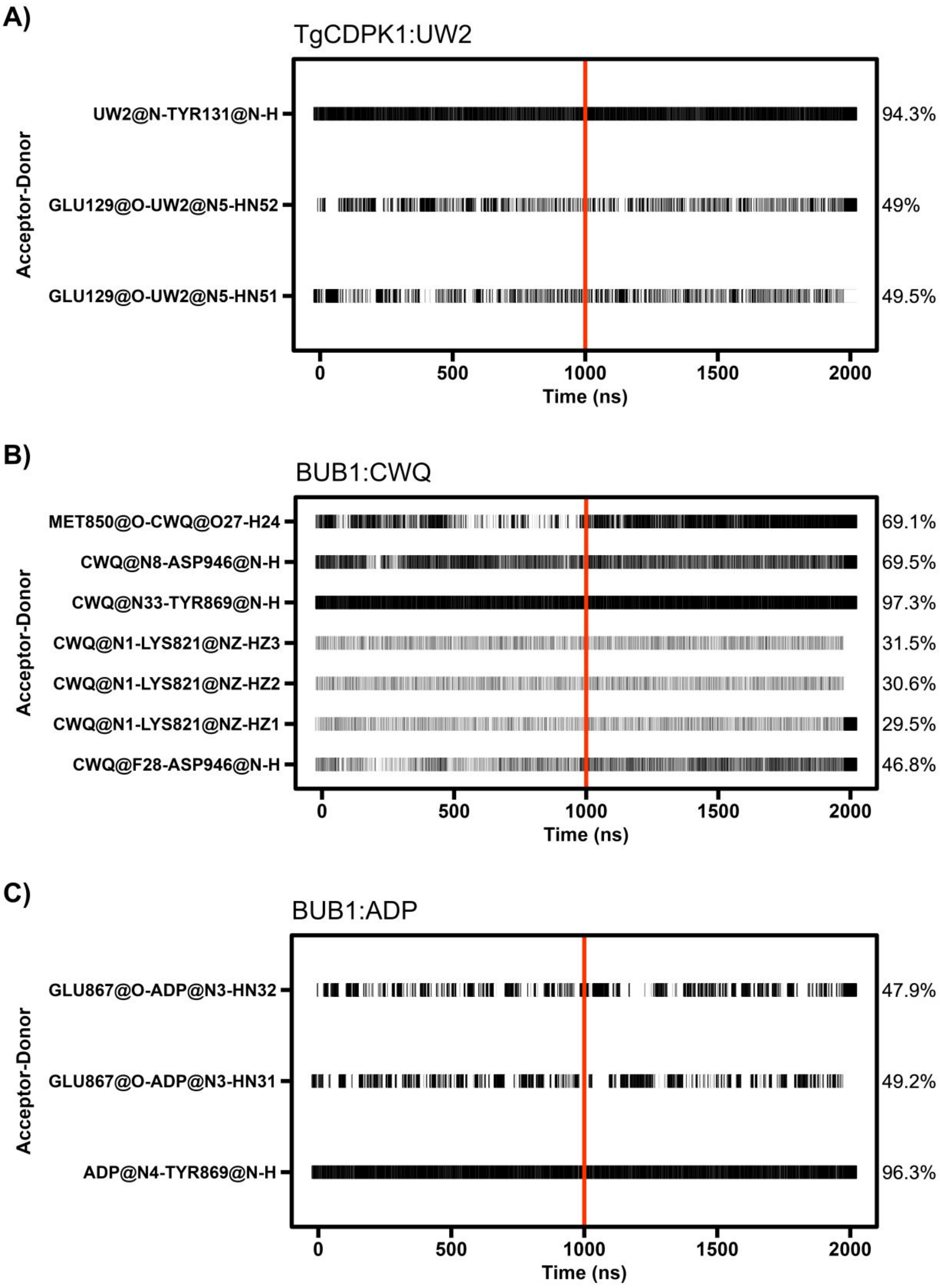

Figure S3. Hydrogen bond analysis of TgCDPK1 and BUB1 complexes with their respective ligands. Hydrogen bond occupancy plots are shown for (A) TgCDPK1:UW2 complex, (B) BUB1:CWQ complex, and (C) BUB1:ADP complex. The occupancy of each hydrogen bond is indicated on the right axis, with higher values indicating more stable interactions. Calculations were performed over the entire 1 μs trajectory for each system. The analysis revealed highly conserved hydrogen bonds with tyrosine residues (Tyr90 in TgCDPK1, Tyr137/135 in BUB1) across all complexes, with occupancies exceeding 90%. TgCDPK1:UW2 displays alternating interactions with Glu88, while BUB1:CWQ exhibits additional stable bonds with Asp214 and Met118. The BUB1:ADP complex shows a distinct pattern involving dual interactions with Glu133. These varying interaction patterns highlight the unique binding modes of each complex and suggest mechanisms for selective recognition.

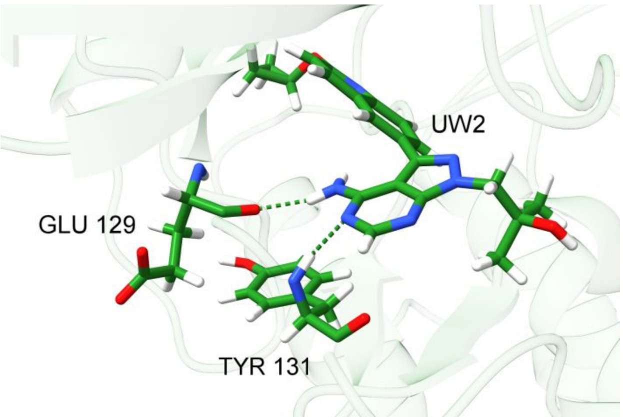

Figure S4. Hydrogen bond interactions in the TgCDPK1 complexed with UW2. Dashed lines represent the hydrogen bonds observed in the simulation.

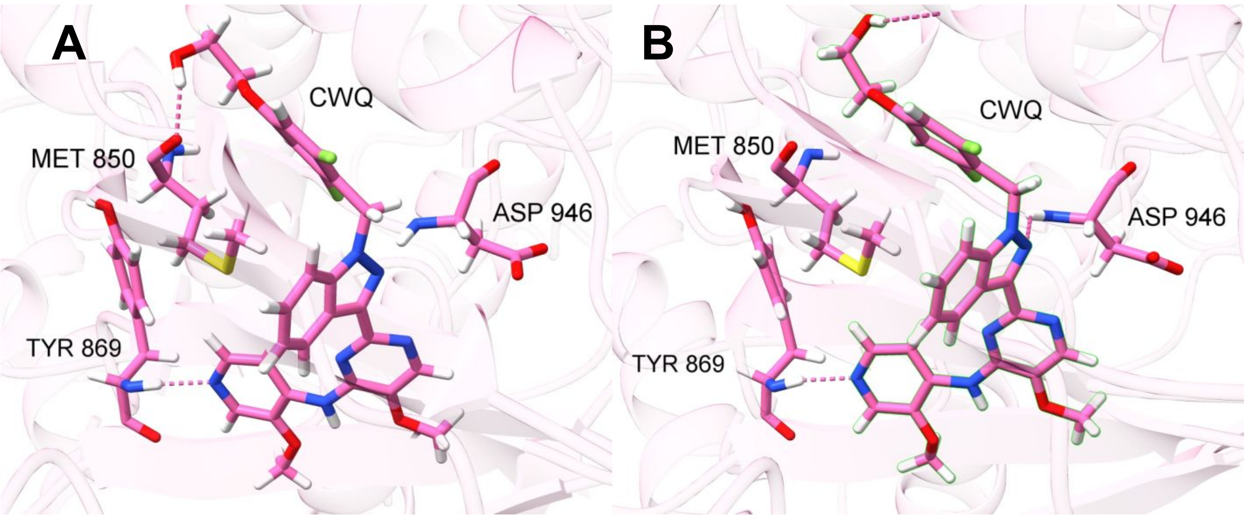

Figure S5. Hydrogen bond interactions in the BUB1 complexed with CWQ. Figures A and B display different orientations of the binding pocket, highlighting key hydrogen bonding interactions stabilizing the ligand CWQ. Dashed lines represent hydrogen bonds detected during the molecular dynamics simulation.

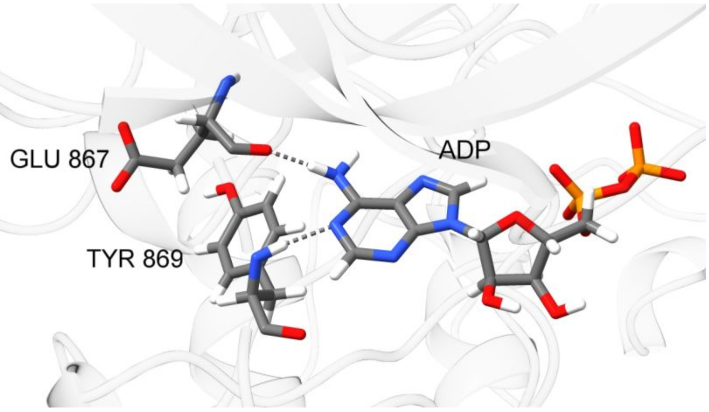

Figure S6. Hydrogen bond interactions in the BUB1 complexed with ADP. Dashed lines represent the hydrogen bonds identified in the molecular dynamics simulation.

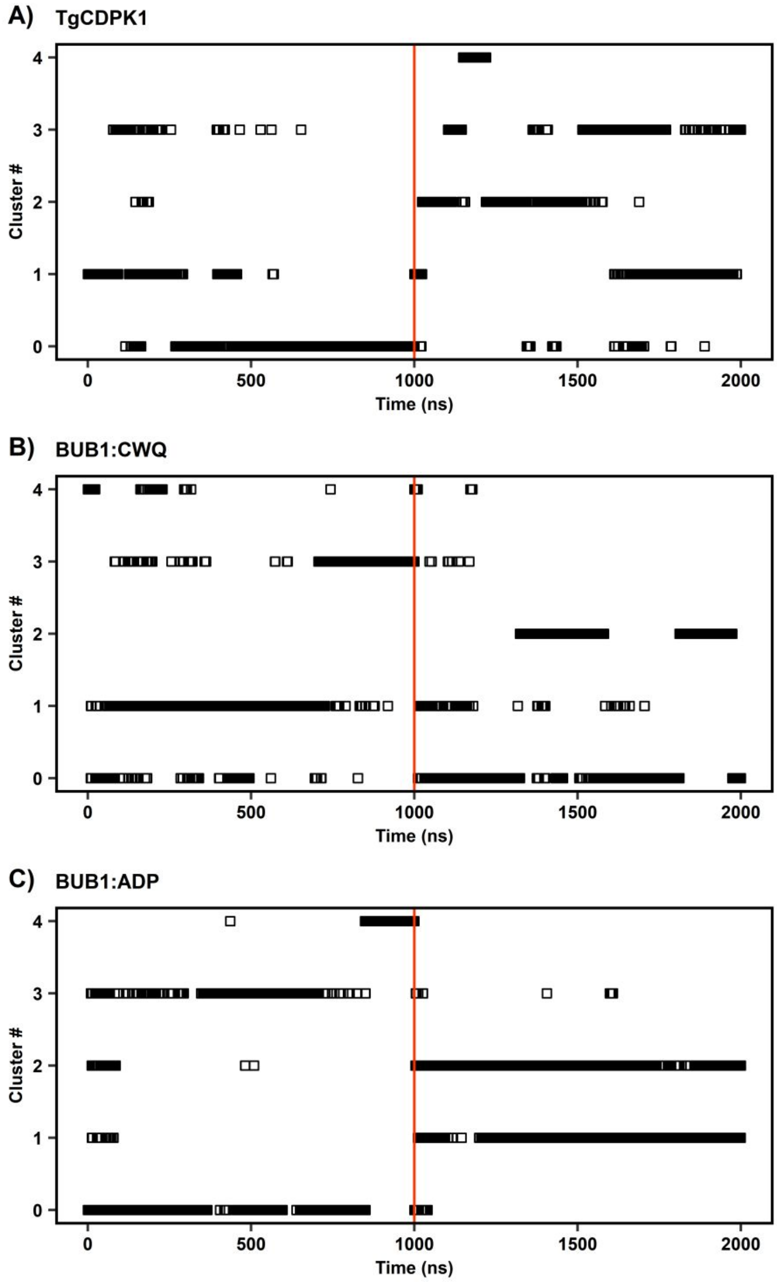

Figure S7. Cluster analysis of ATP-binding site conformations over 2000 ns MD simulations. (A) TgCDPK1, (B) BUB1 complexed with CWQ, and (C) BUB1 complexed with ADP. Five clusters were identified per system using RMSD-based k-means on residues within 5 Å of the ligand. Black bars indicate the presence of each cluster over time; red line marks 1000 ns. Cluster centroids were selected as representative structures for virtual screening.

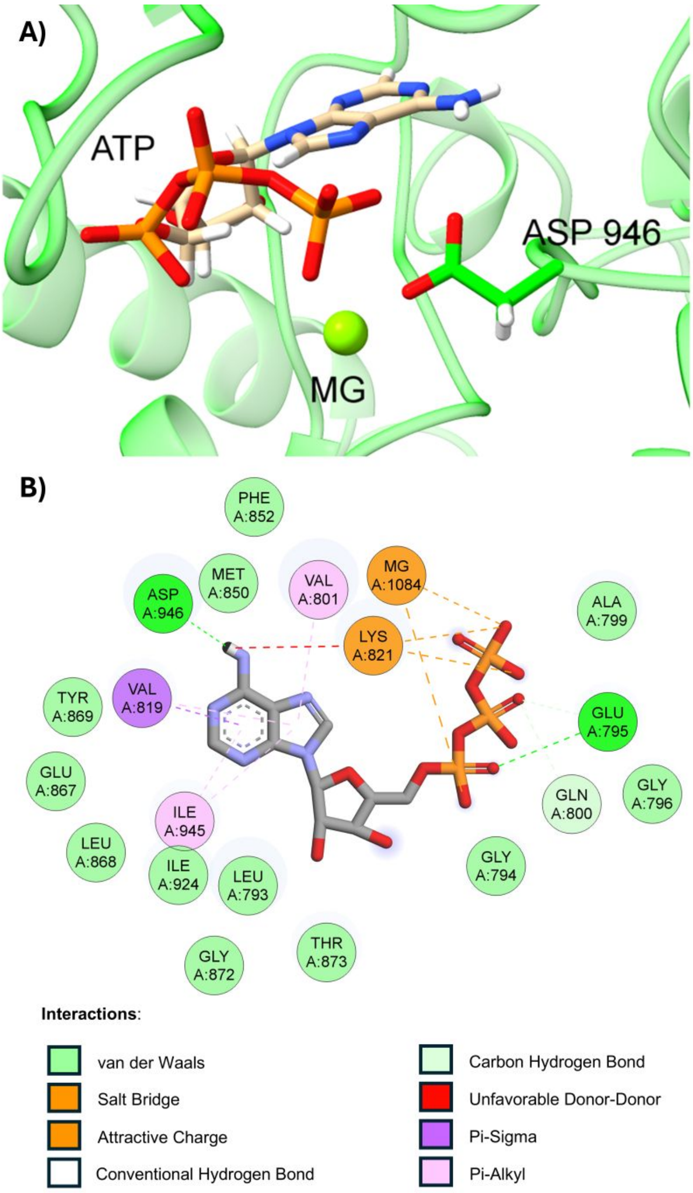

Figure S8. Coordination of ATP by Mg^{2+} and Asp946 in BUB1. (A) Molecular representation of the ATP-binding site in BUB1, highlighting the proximity of Asp946 and the Mg^{2+} ion to the phosphate groups of ATP. The geometry suggests a coordination network stabilizing the molecule. (B) 2D schematic interaction map of frame 4 complexed with ATP showing key contacts between ATP and surrounding residues, including attractive charge interactions with Lys821 and Mg^{2+}, and hydrogen bonding with Glu795 and Asp946. This coordination supports ATP stabilization within the active site.
